# Effects of human impacts on habitat use, activity patterns and ecological relationships among medium and small felids of the Atlantic Forest

**DOI:** 10.1371/journal.pone.0200806

**Published:** 2018-08-01

**Authors:** Paula Cruz, María Eugenia Iezzi, Carlos De Angelo, Diego Varela, Mario S. Di Bitetti, Agustin Paviolo

**Affiliations:** 1 Instituto de Biología Subtropical, CONICET-Universidad Nacional de Misiones (UNaM), Bertoni, Puerto Iguazú, Misiones, Argentina; 2 Asociación Civil Centro de Investigaciones del Bosque Atlántico, Bertoni, Puerto Iguazú, Misiones, Argentina; 3 Facultad de Ciencias Forestales, Universidad Nacional de Misiones (UNaM), Misiones, Argentina; Universidade Federal de Minas Gerais, BRAZIL

## Abstract

Competition theory and niche theory suggest that two morphologically similar species may coexist by reducing the overlap of at least one dimension of their ecological niche. The medium and small Neotropical felids are an interesting group of carnivore species for studying intraguild competition. Due to differences in size it is expected that the larger ocelot exert strong interference competition on the smaller felids (southern tiger cat, margay and jaguarundi); which, in turn, may exert exploitative competition among themselves. Moreover, landscape changes due to human activities may alter these interspecific interactions. We studied the habitat use and the spatial and temporal interspecific relations of the medium and small Atlantic Forest felids, in a landscape with different levels of anthropogenic impact. We estimated the detection probability, and occupancy probability of these cats and whether these parameters are affected by environmental and anthropogenic variables or by the estimated occupancy and detection probability of the ocelot. We estimated the overlap in daily activity patterns between pairs of the four species and changes in their activity in response to anthropogenic impact. We also studied the potential changes that may have occurred in the daily activity of the small felids in relation to ocelot's occupancy probability. The probability of habitat use of the small- and medium-size felids was negatively associated to the intensity of landscape use by humans. Co-occurrence models indicated that the probability of habitat use by southern tiger cats decreased with ocelot occupancy probability. This effect was higher as human disturbance increased. Moreover, the ocelot and the southern tiger cat became more nocturnal in sites with higher human access, suggesting that they may be temporally avoiding encounters with humans or dogs. Conservation of medium and small felids in the Atlantic Forest depends not only on the establishment and implementations of protected areas but also on the management of human's land uses.

## Introduction

Interspecific or intraguild competition is one of the major determinants of community diversity [1]. Competition is stronger as eco-morphological similarity or phylogenetic proximity among competing species increase, and niche theory predicts a limit on the number of species that can coexist in a community [2]. To coexist, species must reduce their overlap in at least one dimension of their ecological niche [3].

One of the possible outcomes of interspecific competition is habitat segregation. Larger, generally dominant, species may exclude smaller or subordinate ones from their territory through interference competition [4–6]. Smaller or subordinate species are usually displaced into suboptimal habitats, such as degraded and less productive environments or those with greater anthropogenic impact [4–6]. Another possible outcome is temporal segregation, that may occur when a subordinate species involved in a competitive relationship adjusts its daily activity pattern to avoid encounters with a dominant species [7].

Competition between species may be by exploitation or by interference. Exploitative competition occurs when one organism consumes resources which are thus unavailable for others [2]. Interference competition occurs when one organism reduces other's ability to make use of resources, usually by means of agonistic or aggressive behaviors [2]. An extreme form of interference competition is intraguild killing [8, 9]. Intraguild killing occurs more frequently when the body weight ratio between competing species (major species/minor species) is between 2.0 to 5.4, and among hypercarnivores of the same taxonomic family [9].

The medium and small Neotropical felids are an ideal group of carnivore species for studying intraguild competition. The ocelot (*Leopardus pardalis*, 6.6–18.6 kg), the jaguarundi (*Herpailurus yagouaroundi*, 3.0–7.6 kg), the margay (*Leopardus wiedii*, 2.3–4.9 kg) and the southern tiger cat (*Leopardus guttulus*, 1.7–3.5 kg), are morphologically very similar (especially the jaguarundi and margay, [10]) and three of them belong to a monophyletic clade of relatively recent radiation (the *Leopardus* sp group, [11–13]). In addition, they have overlapping geographic distributions in the Neotropics [14] and they have similar diets when they coexist at the same location [15–17]. Regarding activity patterns, the ocelot is mostly nocturnal, the jaguarundi is strictly diurnal, the margay is strictly nocturnal, and the southern tiger cat is cathemeral, with the potential to accommodate its activity to that of their competitors [18–20].

Due to the small morphological differences existent among the three smaller cats (see [18]), it is expected that they will exert strong exploitative competition among themselves, and that the larger ocelots will exert strong interference competition, or even intraguild killing, on the smaller felids (the "pardalis” effect, [21, 22]). However, recent studies did not find conclusive evidence that the ocelot exerts a competitive effect on the small felids or affects their habitat use [20, 23].

Through the study of co-occurrence patterns between species, it is possible to identify potential ecological relationships and to understand the role played by these interactions in determining spatial heterogeneity in habitat use within a guild [4]. Occupancy models have been extended to study the patterns of co-occurrence of species and to explore competitive relationships between them [24, 25].

Landscape changes due to human activities may alter the distribution of species, their abundance, and interspecific interactions [26, 27]. Habitat conversion may decrease the availability of resources and may lead to the concentration of species on fragments of native environment, increasing the potential for competition and the chances of direct or indirect encounters (e.g., odor marks) with competing species [27]. Therefore, landscape changes may lead to an increase in competitive relationships among species.

In Argentina, the Atlantic Forest has lost around half of its original extension [28]. Deforestation is mainly due to the conversion of the native forest to large scale pine and eucalyptus monoculture plantations, and to small and medium scale agriculture and livestock farming. In this region, even though the medium and small felids coexist, the ocelots are more abundant in well protected sites while the margay and the southern tiger cat use more disturbed sites, where poaching is common, native forest is under selective logging, and exotic tree plantations are interspersed in the landscape [18]. This pattern of habitat use may result from interference competition exerted by the ocelot on the smaller felids.

The aim of this paper was to study the patterns of habitat use and the spatial and temporal interspecific relations of the medium and small Atlantic Forest felids, in a landscape containing sites with different levels of anthropogenic impact (e.g., continuous forest, forest fragments, pine plantations). We tested if the occupancy probability of felids is negatively affected by human impact, expecting ocelots to be more sensitive than the small felids. We also evaluated if the patterns of habitat use and the daily activity patterns of the smaller cats were affected by the occupancy probability of ocelots. With the use of occupancy models we estimated the probability of detection and occupancy and whether these parameters were affected by environmental and anthropogenic variables. We used two species occupancy models to analyze co-occurrence patterns between ocelots and the smaller felids. We estimated the overlap in daily activity patterns for all pair-wise comparisons of the four felids and analyzed changes in the activity due to anthropogenic impact. We also analyzed changes in the daily activity pattern of the small felids in relation to ocelot’s occupancy probability.

## Materials and methods

### Study area

**Fig 1:**
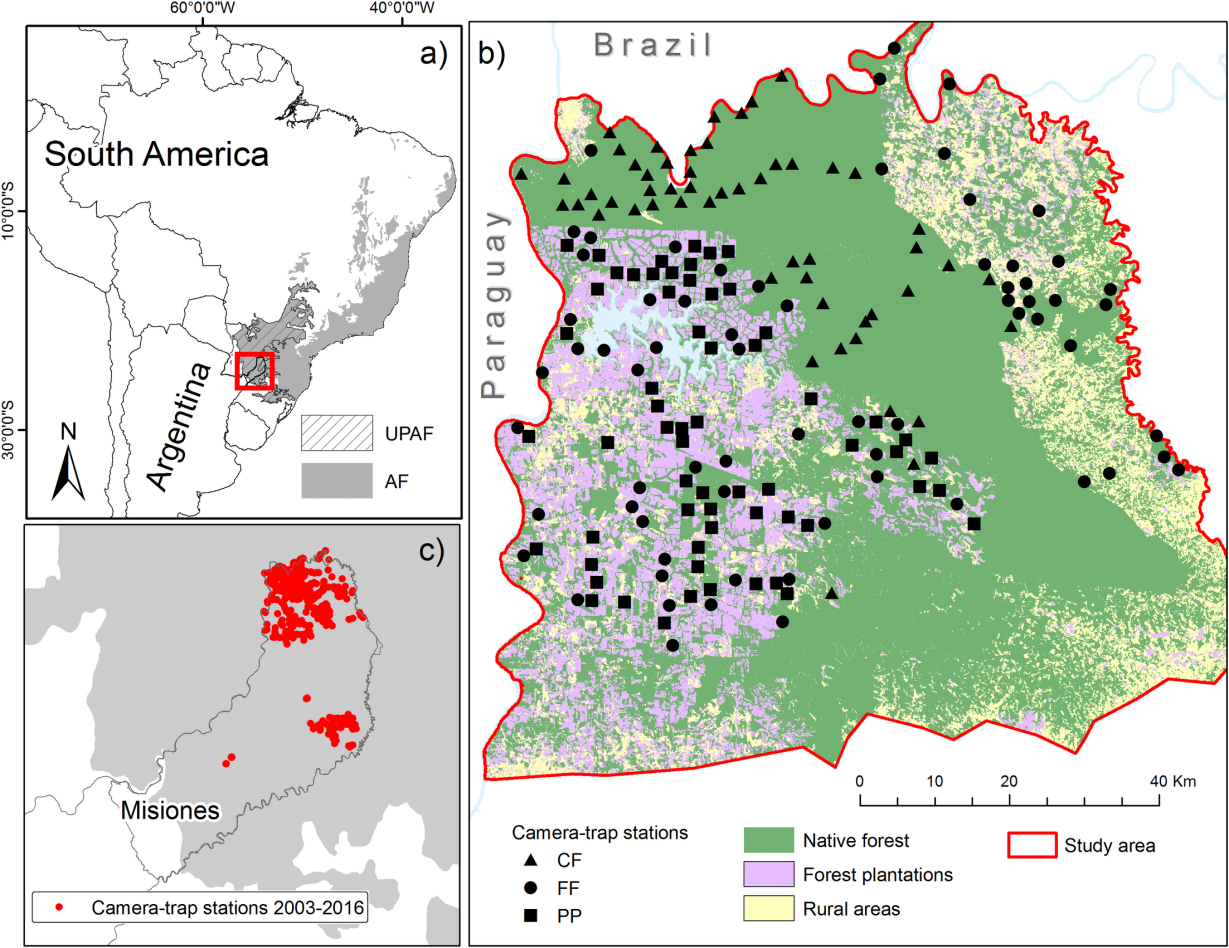
Study area. a) Location of the Upper Parana Atlantic Forest (UPAF), Atlantic Forest (AF) and Misiones province, Argentina (in red). b) Location of the camera-trap stations during a survey conducted in the north of Misiones province between May 2013 and December 2014. The cameras were placed at continuous forest (CF, N = 53), fragmented forest (FF, N = 69) and pine plantations (PP, N = 62). c) Camera-trap stations placed in Misiones province between 2003 and 2016 (N = 586).

We conducted this study in the province of Misiones, Argentina (Fig 1A; 54°15'30.60"W, 25°55'52.32"S). The area contains the world's largest continuous fragment of Upper Parana Atlantic Forest, shared by Argentina and Brazil, and still contains the complete regional native mammal assemblage [29]. Mean monthly temperatures vary between 17 and 22° C and annual rainfall is about 2000 mm with no marked dry season. Seasonality occurs in temperature and day length [30]. This region is covered by a semi-deciduous subtropical forest and is one of the 15 regions that conforms the Atlantic Forest complex. The Atlantic Forest contains high diversity of plants and animals [31] and high levels of endemism, including the southern tiger cat [32]. Is considered one of the “hottest hotspot” of the world [33], and also one of the world's most endangered ecoregions, having lost >94% of its original area [34].

### Data collection

We conducted a camera-trap survey between May 2013 and December 2014 using Reconyx HC500 (manufactured in Holmen, WI, USA) camera-traps in the north of Misiones province. The study area comprises a central large block of continuous native forest (2683 km^2^; Fig 1B), that includes several public and private protected areas (Iguazú National Park, Urugua-í Provincial Park, Urugua-í Wildlife Reserve, etc.) and unprotected areas, with different histories of logging and poaching.

The west part of the study area is mainly dominated by pine (*Pinus taeda*) plantations. Planting density is about 1670 trees per ha, and the stands are neither pruned nor thinned. As a consequence, most of these monoculture pine plantations have a dense canopy cover and scant vegetation undergrowth [35]. Embedded within this pine plantation matrix there are remnants of native forest of different sizes and degree of connectivity with the continuous native forest (Fig 1B). Towards the east, the landscape is dominated by a matrix of rural properties dominated by small-scale agricultural production (yerba mate, tobacco, tea, maize), and small to medium-sized pastures with cattle or swine. There are also fragments of native forest along this matrix with different size, degradation level, and connectivity, and some small public protected areas and private reserves (Fig 1B).

We surveyed three landscape conditions that correspond to different types of human intervention: 1) continuous forest areas, mainly protected areas (CF), 2) forest fragments and strips immersed in a pine plantations matrix or in a rural matrix (FF), and 3) pine (*Pinus taeda*) plantations (PP). The selection of the sites sampled was randomized, using a 2x2 km grid superimposed on the study area, which determined that the sampling stations have a minimum distance of 2 km from each other. Due to access restriction, only accessible grids (those no more than 500 m from a road or trail) were eligible for being surveyed (88% of the potential grids).

We placed 184 surveying stations, 53 in CF, 69 in FF, and 62 in PP (Fig 1B) distributed in a study area of about 3800 km^2^. Each sampling station consisted of a single camera-trap placed at the base of a tree at about 40 cm from the forest floor. Since we only had 36 camera traps available at the beginning of the survey, we allocated 12 camera traps to each of the three landscape conditions during 45–50 continuous days and then we switched cameras to another randomly selected location. Throughout the survey some cameras stopped functioning and some others were stolen with the consequence that less sites where surveyed during each surveying round. Camera-trap stations were settled inside the forest or tree plantation stand, off-roads and trails. The cameras were active on average for 49.8 continuous days (range 18–123 days). Sampling effort (the summation of the number of days each camera trap station was active) totalized 9171.8 trap days.

To characterize forest and understory structure and composition, at each sampling station we performed four *in situ* vegetation measurements at 10-m from the camera trap, towards each of the four cardinal points (N = 4 points). At each point, we identified the presence and abundance of several indicator plant species, we used the punctual interception method [36] to assess understory structure and density and the point-centered quarter method [37] to estimate tree density and basal area. From these measurements and using Principal Component Analysis (PCA) we derived a single variable (the first PCA axis) that characterizes vegetation structure (for details see S1 Table).

In addition to the camera-trap survey described above, we added data from systematic and non-systematic surveys performed by our team between 2003 and 2016 at different sites within the Misiones province (Fig 1C) to study the felids daily activity patterns. The systematic surveys (N = 7) were developed between 2003 and 2014, mainly to estimate jaguar (*Panthera onca*), puma (*Puma concolor*), and ocelot densities. Three hundred and fifty two stations were active for a total of 21,189 camera-trap days. Sampling stations for these surveys consisted of two camera traps facing each other at both sides of unpaved roads and trails (see [38–41]). During non-systematic surveys, samplings stations (N = 48) did not follow a specific methodology, but mostly consisted of a single camera trap deployed on unpaved roads and trails. To avoid pseudo-replication a period >1 hour had to pass for two successive photographs of each felid to be considered independent records [42]. Overall, for the analysis of the daily activity patterns we used data from 586 trap stations.

### Habitat use and co-occurrence data analysis

To analyze the patterns of habitat use and the spatial relationships of the medium and small felids, we used Single-species Single-season and Two-species Single-season Occupancy Models, with a likelihood-based approach. The occupancy models estimate the probability that an area (the camera-trap location) is occupied or used by a species (ψ), and also the probability of detecting (*p*) the species at each sampling station given that the station is occupied by the target species. These models allow incorporating covariates to study their effect on the occupancy probability (ψ) and the detection probability (*p*) of a species [43]. The probability of detection (*p*) is estimated by repeated sequential visits to a specific area (sampling occasions, [24]). In our case we divided the sampling period in 13 successive sampling occasions of 9 continuous days each.

Estimates of the occupancy probability (ψ), and the probability of detection (*p*) can be influenced by the species’ abundance and some authors consider ψ as a proxy of abundance [24, 44]. A fundamental assumption of occupancy analysis is that populations are closed (i.e. occupancy state in each site does not change during survey) [24]. However, this assumption could be relaxed if changes in a population occur at random, and also if ψ is interpreted as the probability that the species uses the area where the sampling station is located [24], as in our study. In fact, given the extremely small size of the effectively sampled area (i.e. the area covered by the infrared sensor of the camera trap) in relation to the size of the home range of the target species, ψ should be interpreted as the probability that the camera trap station is located within the home range of at least one individual of the target species [45, 46]. In this sense, the estimate of ψ for a particular camera trap station could also be interpreted as the probability that the camera trap station is located in an area that constitute habitat that is being used by the target species.

#### Environmental and anthropogenic variables

We performed a PCA based on the correlations of the standardized *in situ* vegetation measurements using Infostat program [47]. Values of axis 1 of this PCA were used to represent the vegetation structural complexity, with positive values representing sites with higher structural complexity (native forest sites with higher diversity of strata and species), and negative values indicating sites with scant understory vegetation (pine plantations).

Using the *Focal Statistics* tool in ArcGIS 10.1 we estimated the percentage of native forest remaining in a circle with a 2-km radius centered at the camera-trap station. This variable is representing the abundance of forest in the area around the camera trap location. The value of 2 km approximates the radius of an ideal circular home range of a female of these species in the Atlantic Forest [48, 49], and may approach the scale at which the species perceives the landscape [50–52].

As an approximate measure of anthropogenic impacts we used the variable "human cost of access" (see [53]) which estimates the time it would take to a human being to access each sampling site from the nearest urban location. Accessibility will depend, among other things, on the presence of roads, topography, vegetation type, and the legal level of protection of the areas. It is expected that this variable correlates with the impact of different activities such as human presence, poaching, timber harvesting, abundance of dogs and other domestic animals, among others [53, 54].

As a rough estimate of relative food abundance we also estimated the recording rate (records/100 days) of the potential main prey of these felids. For the three small felids, we considered as main potential prey all the small rodents (Subfamily Sigmodontinae) recorded by the camera traps. For the ocelot we consider as main prey the sigmodontines and the agouti (*Dasyprocta azarae*). This prey selection was based on a study of the diet of these felids at the study area [55]. Finally, to assess if there was collinearity among the continuous independent variables we performed Spearman correlations using the Infostat program [47]. We considered a value of |rho| = 0.65 as a threshold to differentiate variables with strong correlation from variables with weak correlation.

#### Single-species occupancy models

To evaluate changes in the probability of habitat use (ψ) of the species, we used five covariates: landscape condition (CF, FF, PP; categorical covariate with PP being the reference (intercept) group), human cost of access, vegetation structure (values on axis 1 of PCA), percentage of native forest in a 2-km radius, and prey recording rate. These covariates were considered to have potential impact on ψ according to *a priori* hypotheses (S2 Table). Since camera trap detection probability (*p*) could be influenced by understory cover we used, as a proxy, the number of contact points of the vegetation on the rod section from 0 to 1 m (N = 4 measurements per station; see Data collection and S1 and S2 Tables).

We run 64 models, resulting from the combination of all the covariates, since our main interest was to know the importance of the variables and their effect on ψ and *p* [56–58]. We run the occupancy models using *unmarked* package [59] with the open-source software R 3.1.2 [60]. We analyzed overdispersion of the data through the goodness of fit test of the most parameterized model [61]. Models were ranked by their Akaike's Information Criterion adjusted for small sample size (AICc) ascending value. We analyzed the relative importance of each covariate using their cumulative AICc weights (w+) [56, 57]. We also used the confidence intervals (CI) of each covariate present in the best ranked models (i.e. models with ΔAICc≤2) to evaluate its importance [57, 62].

Since there were no recaptures at any station on different samplings occasions for the jaguarondi and there were only two recaptures for the margay, it was not possible to run occupancy models for these felids. For these species we performed a Fisher's Exact Test [63] using R 3.1.2 to test for a possible association between their records and the landscape condition (CF, CC, PP).

#### Two-species occupancy models

The occupancy models for two species analyze the co-occurrence of species considering their detection probability [24, 44]. These models allow the analysis of asymmetric ecological interactions (e.g. predator-prey) and/or behavioral responses among co-occurring species. One of the species (A) is defined as the dominant species and the other species (B) as the subordinate one [24, 25]. The model estimates the occupancy probability of the dominant species (ψA), the occupancy probability of the subordinate species when the dominant species is present (ψBA), and when it is absent (ψBa), and the unconditional occupancy probability of the subordinate species (ψB; estimated following [25]). Using these parameters, a Species Interaction Factor (SIF) is calculated. If the SIF is >1, the species co-occur more than expected by chance (species A facilitates the presence of species B). If the SIF = 1, the species do not interact. Finally, if the SIF <1, the subordinate species avoids the dominant species. These models also allow analyzing whether the presence or detectability of species A can influence the detectability of species B, and vice versa [25].

To run these models, we used the PRESENCE 6.2 program [64], and the "ψBa /rBa parameterization" option. Due to the scarce data obtained from jaguarundi and margay, it was only possible to run these models for the ocelot- southern tiger cat pair. We considered the ocelot as the dominant species (A), and the southern tiger cat as the subordinate (B). As covariates for modeling occupancy and detection probability we used the covariates that had a strong evidence of an effect on these parameters (ψ and *p*) in the single-species models: the landscape condition and the cost of access for ψ and no covariates for *p* [25, 65, 66].

Models were ranked by their AICc ascending value, and the best models were selected using the ΔAICc≤2 criterion [56]. To calculate the Species Interaction Factor (SIF), the unconditional occupancy probability for each species (ψA and ψB) and the occupancy probability for species B conditional on either the presence or absence of species A (ψBA and ψBa) at each sampling station and the CI for each parameter we used R 3.1.2 and the *delta method* function of the msm package [25, 60, 65–67].

### Activity data analysis

We used kernel density functions [68] to compare the daily activity patterns among felids and quantify their similarity with the *overlap* R-package [69, 70]. Time of day is used as a random circular variable in kernel density estimates [71]. This analysis estimates an overlap coefficient (Δ), which varies from 0 (no overlap) to 1 (total overlap). To statistically evaluate whether the activity patterns of each felid were different from each other, we used the Mardia-Watson-Wheleer test [72].

In order to evaluate if the small felids modify their activity pattern in relation to the occurrence of ocelots, we used the ocelots' occupancy models to estimate its occupancy probability at each sampling station. We classified stations as those with low ocelot's occupancy probability (ψ = 0–0.40) and those with high occupancy probability (ψ = 0.60–0.99). Stations with medium occupancy probability (ψ = 0.41–0.59) were discarded from this analysis. Since ocelots are mostly nocturnal [18], we expected that at sites with high ocelots’ occupancy probability small felids will switch their daily activity patterns to reduce overlap with the ocelot (S3 Table), with the southern tiger cat becoming more diurnal when compared with sites with low probability of ocelot occupancy. We also evaluated whether the felids modify their activity patterns according to the level of anthropogenic impact. For this, we georeferenced all the stations using ArcGis 10.1 and estimated for each one its "human cost of access" [53]. Finally, we classified daily activity records as belonging to sites with low (0–1) or high (1.5–6.0) cost of access. Stations with medium cost of access (1.1 to 1.4) were discarded from this analysis. Because human activities and poaching are mainly carried out during the day we expected that, at sites with higher anthropogenic impact, felids will have more nocturnal activity than at sites with lower human impact (i.e., with a higher cost of access). We expected a greater response for the southern tiger cat (S3 Table). We performed a Mardia-Watson-Wheeler test [72] to assess whether the daily activity patterns differ between contrasting situations. All statistical analyses were implemented in R 3.1.2 [60].

### Ethics statement

The Ministry of Ecology of Misiones province, National Parks Administration of Argentina and private owners provided permission to conduct this project in their respective jurisdictions. During this research, animals were remotely recorded in their natural environment and none of them were captured or handled. Therefore, given our study protocols there were no particular requirements by governmental agencies aimed at minimizing potential impacts of our research on animals or the environment.

## Results

### Patterns of habitat use

We obtained 4923 mammal records, only 120 of which were from the studied felids: 48 of ocelot, 14 of jaguarundi, 10 of margay, 41 of southern tiger cat, and 7 of small felids identified at the level of genus (*Leopardus* sp., Table 1). The latter were discarded from the analysis.

**Table 1:** Frequency of camera trap records and number of stations with presence (in parentheses) of medium and small felids at different landscape conditions in northern Misiones province, Argentina, where a camera trap survey was conducted between May 2013 and December 2014.

Felids	Continuous forest (N = 53 stations)	Forest fragments (N = 69 stations)	Pine plantations (N = 62 stations)
**Ocelot**	38 (21)	6 (5)	4 (3)
**Jaguarundi**	6 (5)	8 (8)	0 (0)
**Margay**	2 (2)	8 (6)	0 (0)
**Southern tiger cat**	6 (6)	24 (14)	11 (8)
***Leopardus* sp.**	2 (2)	3 (3)	2 (2)
***Total***	*54*	*49*	*17*

**Fig 2:**
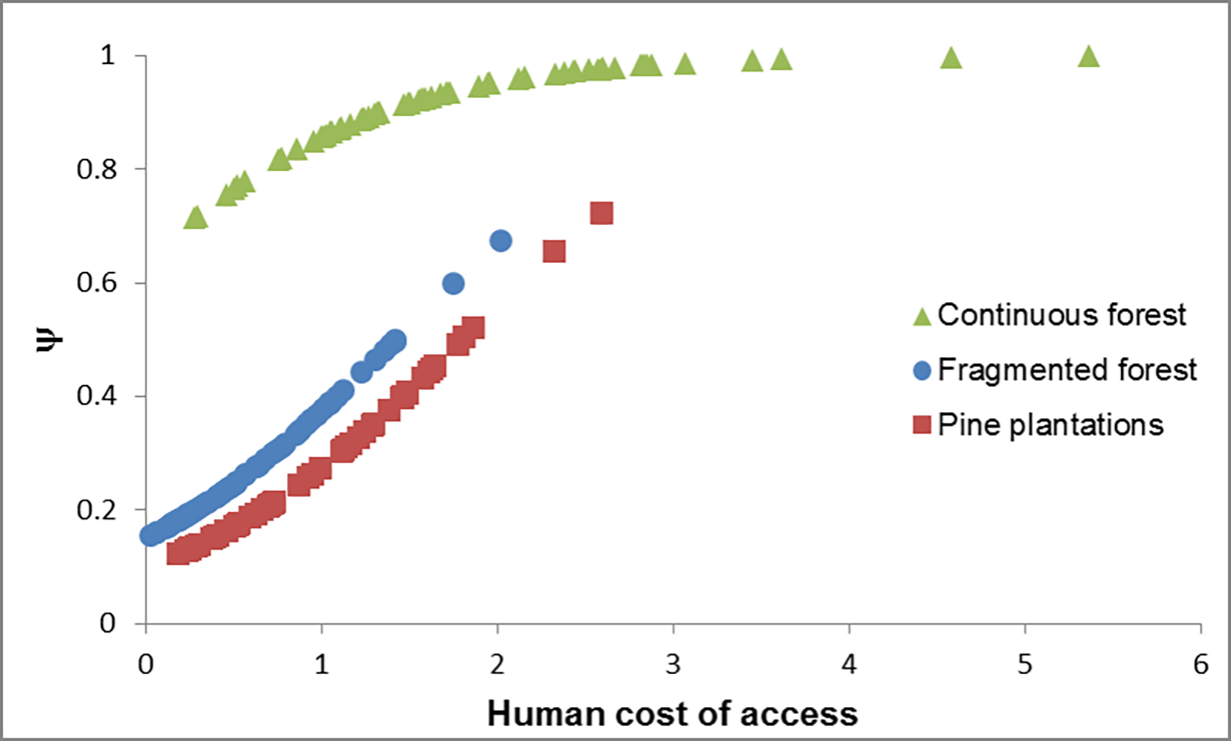
Effect of the human cost of access and habitat type on the occupancy probability of ocelots (ψ) in northern Misiones province, Argentina.

For the ocelot the goodness of fit test revealed no overdispersion of the data (χ^2^ = 906.67; P = 0.46; c-hat = 0.12). The null model was the last ranked model (with a ΔAICc = 27.23; S4 Table), denoting the importance of the alternative models. The cumulative AIC weight (w+) was 0.82 for landscape condition and 0.77 for human cost of access (S5 Table). These covariates were the only ones present in the best ranked model and their 95% CI did not overlap with 0. All other covariates had w+<0.50. For these covariates the 95% CI overlapped with 0 in all the models with ΔAICc≤2 (S5 Table). In the most parsimonious model that contained the most important covariates (i.e. the best ranked model), the estimated probability of detection of ocelots (*p*) was 0.13 (± 0.03). The occupancy probability (ψ) of ocelots was higher in the continuous forest (CF) than in the other landscape conditions (CF = 0.91 (± 0.07), FF = 0.29 (± 0.12), PP = 0.27 (± 0.14); Fig 2, S1 Fig). In addition, ψ was inversely related to human accessibility (Fig 2).

For the southern tiger cat there was also no overdispersion of the data (χ^2^ = 96.11; P = 0.45; c-hat = 0.96). The null model was among the best ranked models, with a ΔAICc = 0.45 (S6 Table). The cumulative AIC weight (w+) was 0.56 for human cost of access and lower than 0.50 for all other covariates (S7 Table). All covariates had their 95% CI overlapped with 0 (S7 Table).

For the jaguarundi and margay no records were obtained in the pine plantations (PP, Table 1) and results of the Fisher's Exact Test suggest a negative association of the records of these species and this environmental condition (jaguarundi: p = 0.009, margay: p = 0.039).

### Patterns of co-occurrence of ocelot and southern tiger cat

No co-occurrence model had a ΔAICc≤2 from the top ranking one (S8 Table). The first ranked model included cost of access (only for the ocelot) and landscape condition (for both species) as variables (S9 Table). This model suggests that the occupancy probability of the southern tiger cat decreases with the occupancy probability of the ocelot (ψBA = 0.21 ± 0.08, ψBa = 0.87 ± 0.08). The Species Interaction Factor (SIF) was less than 1, with a mean value of 0.47 (95% CI = 0.37–0.58), indicating a negative effect of ocelots on southern tiger cats. The SIF differed by landscape conditions: CF = 0.95 (95% CI = 0.92–0.99); FF = 0.42 (95% CI = 0.20–0.63); PP = 0.06 (95% CI = 0–0.19), suggesting that southern tiger cats avoid ocelots with higher intensity in pine plantations than in continuous forest. The SIF also increased with the cost of access. The probability of detection of southern tiger cats (*p* = 0.07 ± 0.02) did not vary with the presence or detectability of ocelots.

**Fig 3:**
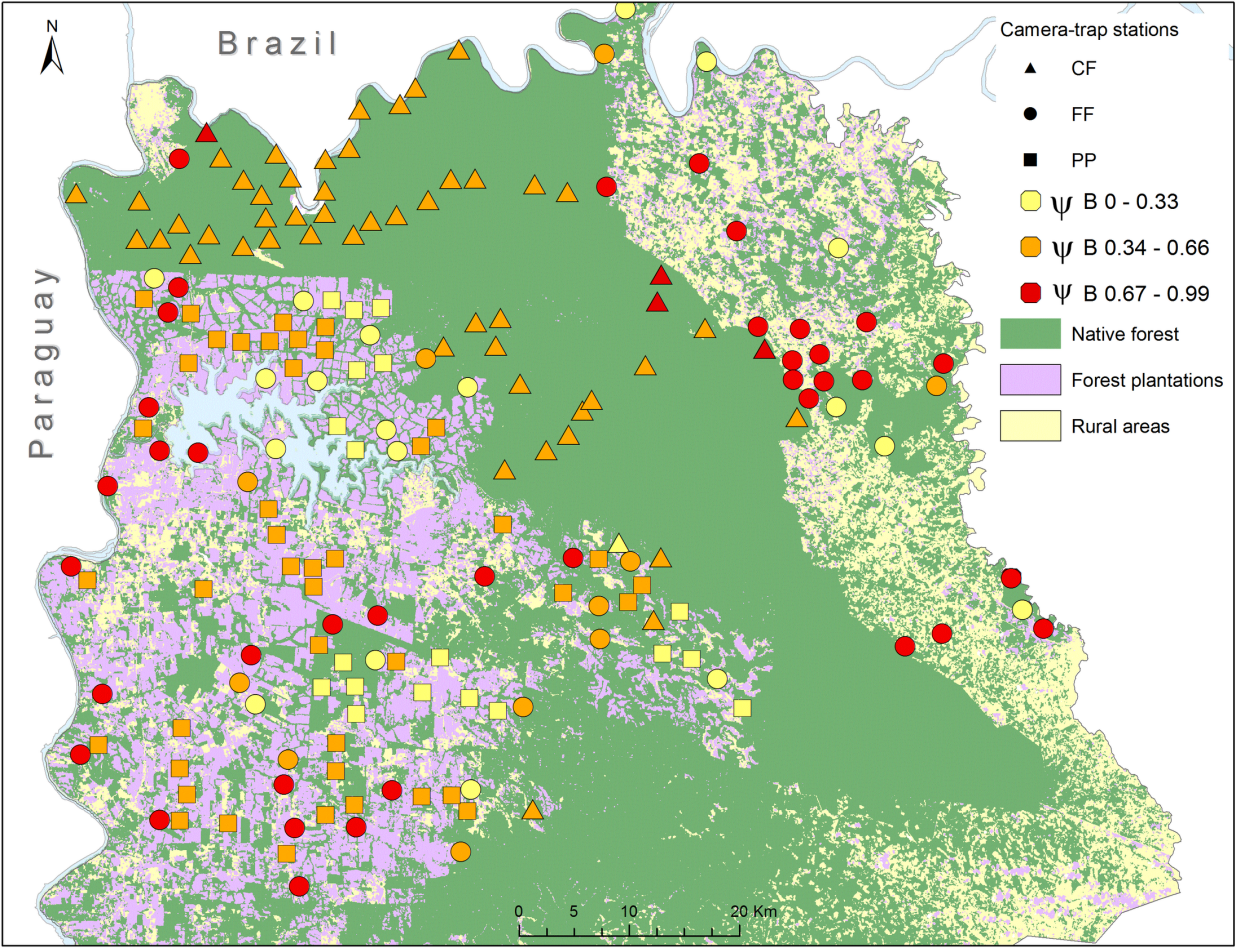
Occupancy probability of southern tiger cats (ψB). Location of the camera-trap stations (N = 184) with low (0–0.33, yellow), intermediate (0.34–0.66, orange), and high (0.67–1.00, red) occupancy probability of southern tiger cats according to the model of co-occurrence with the ocelot. Triangles = stations located in continuous forest, circles = forest fragment stations, squares = pine plantations.

Finally, the unconditional southern tiger cat occupancy probability (ψB) showed no differences among landscape conditions (S10 Table). However, stations located in continuous native forest (CF) had mainly intermediate values of ψB, stations located in fragmented forest (FF) showed highly variable ψB, and stations in pine plantations (PP) showed low and intermediate values of ψB (S11 Table, Fig 3). The stations with higher values of ψB were located in FF with very low occurrence of ocelots (mean ψA = 0.10 ± 0.15 (SD)).

### Daily activity patterns

**Fig 4:**
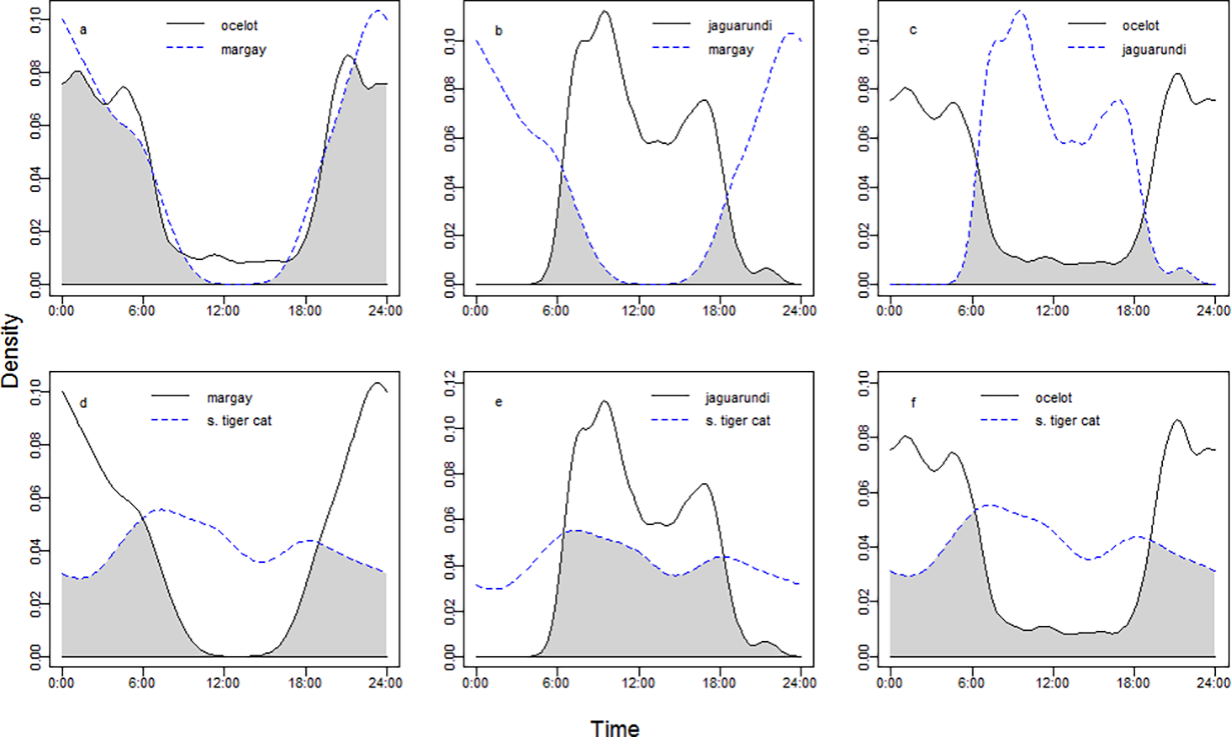
Activity and overlap of the small and medium felids. Temporal activity and degree of overlap (shaded area) among: a) ocelot and margay, overlap coefficient (Δ) = 0.89 with 95% CI = 0.82–0.95; b) jaguarundi and margay, Δ = 0.17 with 95% CI = 0.09–0.25; c) ocelot and jaguarundi, Δ = 0.21 with 95% CI = 0.17–0.25; d) margay and southern tiger cat, Δ = 0.57 with 95% CI = 0.47–0.66; e) jaguarundi and southern tiger cat, Δ = 0.63 with 95% CI = 0.56–0.69; f) ocelot and southern tiger cat, Δ = 0.60 with 95% CI = 0.53–0.66.

We obtained 1784 independent records of activity for ocelots, 213 for southern tiger cats, 155 for jaguarundis and 69 for margays. The greatest daily activity overlap occurred between the ocelot and the margay (Fig 4A), and without statistical differences in their activity pattern (Mardia-Watson-Wheeler test, χ2 = 0.13 df = 2, p = 0.94). The lowest overlap occurred between the jaguarundi and the margay, and between the jaguarundi and the ocelot (Fig 4B and 4C). In both cases, the activity patterns were statistically different (margay-jaguarundi: χ^2^ = 40.81 df = 2, p < 0.001; jaguarundi-ocelot: χ^2^ = 22.21 df = 2, p < 0.001). The southern tiger cat had a similar overlap value with the other felids (Fig 4D, 4E and 4F); with patterns that were statistically different (southern tiger cat-margay: χ^2^ = 43.77, df = 2, p < 0.001; southern tiger cat-jaguarundi: χ^2^ = 27.76 df = 2, p < 0.001; southern tiger cat-ocelot: χ^2^ = 13.43, df = 2, p = 0.001).

**Fig 5:**
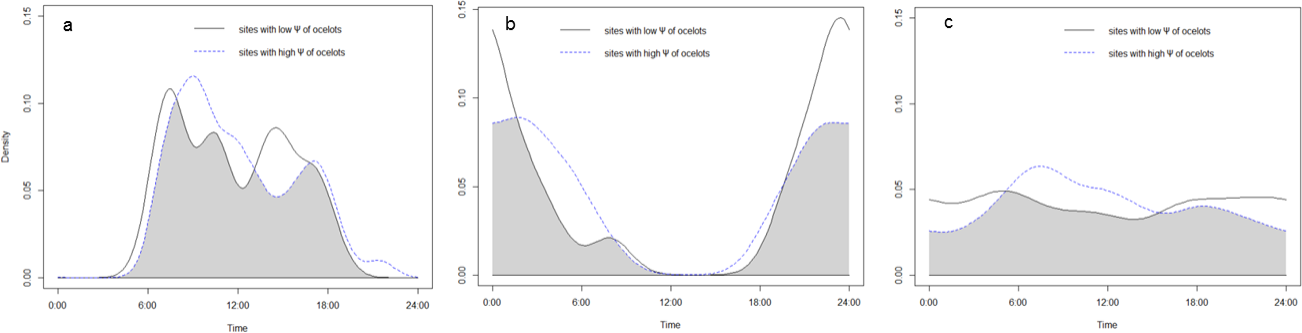
Activity of small felids according to the occupancy probability of ocelots. Activity patterns and overlap between: a) the daily activity pattern of jaguarondis at sites with low ψ of ocelots (N = 31 activity records) vs. the activity at sites with high ψ of ocelots (N = 90 activity records), b) the daily activity pattern of margays at sites with low ψ of ocelots (N = 14 activity records) vs. the activity at sites with high ψ of ocelots (N = 41 activity records), c) the daily activity pattern of southern tiger cat at sites with low ψ of ocelots (N = 72 activity records) vs. the activity at sites with high ψ of ocelots (N = 119 activity records).

**Fig 6:**
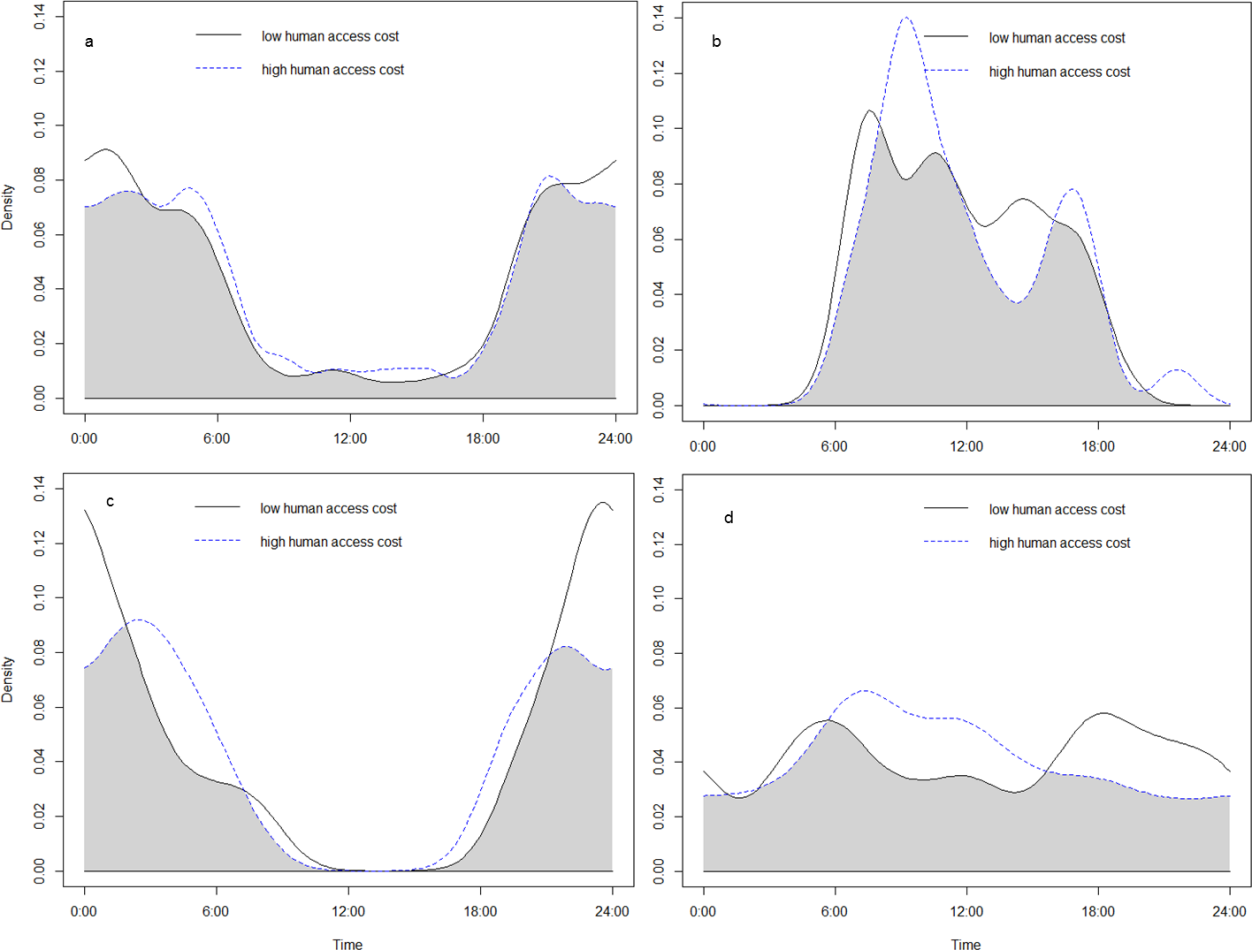
The daily activity pattern of small felids according to human cost of access. Activity patterns and overlap between: a) the activity pattern of ocelots at sites with low human cost of access (N = 481 activity records) vs. the activity at sites with high human cost of access (N = 937 activity records), b) the activity pattern of jaguarundis at sites with low human cost of access (N = 55 activity records) vs. the activity at sites with high human cost of access (N = 32 activity records), c) the activity pattern of margays at sites with low human cost of access (N = 16 activity records) vs. the activity at sites with high human cost of access (N = 25 activity records), d) the activity pattern of southern tiger cats at sites with low human cost of access (N = 79 activity records) vs. the activity at sites with high human cost of access (N = 85 activity records).

None of the three small felids changed its activity pattern in relation to the occupancy probability of ocelots (jaguarundi: χ^2^ = 1.01, df = 2, p = 0.60, Fig 5A; margay: χ^2^ = 2.75, df = 2, p = 0.25, Fig 5B; southern tiger cat: χ^2^ = 4.91, df = 2, p = 0.09; Fig 5C). The southern tiger cat and the ocelot, but not the other two species, became more diurnal in areas with higher human cost of access (ocelot: χ^2^ = 6.37, df = 2, p = 0.04, Fig 6A; jaguarundi: χ^2^ = 0.94, df = 2, p = 0.63, Fig 6B; margay: χ^2^ = 2.8, df = 2, p = 0.25, Fig 6C; southern tiger cat: χ^2^ = 7.27, df = 2, p = 0.03, Fig 6D).

## Discussion

### Habitat use patterns

The patterns of habitat use of the small- and medium-size felids of the Atlantic Forest of Argentina were clearly associated to patterns of landscape use by humans. The occupancy models and the number of records obtained suggested that the sites with the greatest human impact, the pine plantations, were the environments less used by these felids.

The ocelot's occupancy probability was higher in areas of continuous native forest and high cost of access for humans. These results suggest that the ocelot is quite sensitive to habitat changes and anthropic pressures, as was previously reported [38, 42, 73–75]. The lower occupancy of ocelots in areas accessible to humans could be due to the greater presence of domestic carnivores (dogs and cats), which either directly persecute ocelots (dogs) or transmit diseases that negatively affect their populations [76–78]. Domestic dogs negatively influence ocelot’s abundance in other Atlantic Forest regions [75]. Poaching could be another possible factor, since hunting pressure is more intense at more accessible sites [79–82]. Although the ocelot is not currently sought after by poachers in Misiones [29], hunting could have an indirect effect on its abundance, since some of its prey are heavily affected by poaching [83, 84].

The lower ocelot occurrence in fragmented forest has been previously observed in the Amazon [85] and the Atlantic Forest [75]. In the current study, forest fragments with higher habitat use by ocelots were close to the continuous forest (S1 Fig). Many of the sampled fragments were too small to hold populations or even resident ocelots. Individuals recorded at forest fragments are likely to use several of them to meet their requirements, and the presence of ocelots is conditional to the connectivity of the landscape, and closeness to the continuous forest. The lower use of areas with pine plantations is consistent with previous results obtained in the same area [42]. The lower occupancy of ocelots in this structurally simplified environment could result from the lack of shelters to escape from competitors (human, dogs, larger felids) or from less successful hunting due to the lack of shelters.

For the margay and the jaguarundi, the paucity of data precluded the use of occupancy models. However, even though we were not able to control for the probability of detection of these felids in these different environments, independence tests indicate a negative association between the records of these felids and pine plantations. As with the ocelot, this productive activity may have a negative effect on the populations of these felids in the Atlantic Forest due to the reduction on the abundance of their main prey, a lower hunting success due to low availability of hiding places and/or a higher perceived risk due to the lack of refuges to escape from predators. However, these results should be considered with care because they do not account for imperfect detection.

The detectability or the frequency of records of felids in our study was extremely low, especially for margay and jaguarundi. This low detectability (and the low naïve occupancy probability) was expected, especially for ocelots, since camera-traps were not placed on trails or roads, which greatly increase the detection probability of this felid [18, 86, 87]. For the smaller felids the very low detectability could indicate a low abundance of these species in Misiones, since detectability may be related to species abundance [44]. This is also supported by the low number of records obtained in the systematic and non-systematic surveys performed by our team between 2003 and 2016 at different sites within the Misiones province [18, 38, 41, 42, 87], and the low encounter rate of feces for these felids obtained throughout the study area (Paviolo et al. unpublished data). The arboreal habits of the margay may have also contributed to its low detectability [88, 89].

### Co-occurrence patterns

The occupancy probability of the southern tiger cat varied in relation to that of the ocelot, being higher in sites with low or no occurrence of ocelots. These results suggest that the southern tiger cat is very sensitive to the competitive pressure exerted by the ocelot [21]. In addition, the Species Interaction Factor (SIF) suggests that southern tiger cats avoid ocelots, with increasing avoidance as anthropogenic impacts increase. Landscape changes resulting from human activities seem to affect ocelots and southern tiger cats competitive interactions and their mechanisms of coexistence. Ecological theory predicts that interference competition should increase as resources become limited [4]. For example, habitat fragmentation and prey shortage can induce dominant predators to move longer distances to acquire food [90], increasing the encounters chances with subordinate species [4]. Also, changes in vegetation structure (e.g., at pine plantations) could affect the vulnerability of the subordinate species, increasing its visibility or detectability and may also reduce shelter availability and the chances to escape during a predation attempt [91].

Sites with higher southern tiger cats occupancy (ψ = 0.67 to 1) were located mostly in forest fragments with low or no ocelot occurrence. These sites with an intermediate degree of disturbance could thus be playing an important role as strongholds for this small felid. A similar pattern was observed in other areas of Atlantic Forest in Brazil, where southern tiger cats inhabit fragmented forests where ocelots are rare or absent [17, 49], whereas in continuous native forest the ocelot becomes the most abundant felid, as at [92] and our study. This could be the reason why [20] and [23] did not find a spatial avoidance of ocelots by the small felids. These studies covered smaller areas and with smaller forest remnants (up to 350 km^2^). In contrast, our study was developed in a much larger landscape that includes one of the biggest continuous remnants of Atlantic Forest in the world (2,683 km^2^) and an extended area of fragmented forests.

### Daily activity patterns

As it was expected, the four felids tend to have distinct daily activity patterns, with the exception of the nocturnal ocelot-margay pair, which has the greatest overlap in activity. The diurnal jaguarundi has low overlap with ocelots and, specially, with margays. Because jaguarundis and margays have the greatest similarity in trophic morphology among Neotropical felids [10], their contrasting daily activity patterns are likely to result from species sorting or as a behavioral adaptation to reduce interspecific competition [18]. On the other hand, these two felids do not seem to modify their activity patterns in relation to the ocelot occupancy or to different levels of human access. These results and the similarity of their activity patterns across their distribution range [18, 89, 92–97] suggest phylogenetic constraints to modify this behavior.

The ocelot and the southern tiger cat showed greater nocturnal activity in sites with higher human access, suggesting that they may be temporally avoiding encounters with humans or domestic animals [98]. However, the change in the ocelots’ activity was slight when compared to the important shift shown by southern tiger cats (Fig 6) and may have been statistically detected due to the large amount of data used in this analysis (N = 1418 ocelot records).

The southern tiger can adjust its activity pattern to reduce interspecific competition and becomes mostly nocturnal at places where the ocelot, margay and puma are absent [19]. In the current study, the southern tiger cat did not statistically modify its activity with ocelot’s occupancy (even though it showed the expected shift, Fig 5C). However, it became more nocturnal in areas with higher human impacts and where, as a consequence, ocelots were absent or rare. Therefore, we cannot rule out the existence of an additive effect of human impact and ocelot occurrence on the southern tiger cat’s daily activity.

## Conclusion

Interspecific competition is one of the main mechanisms that define guild structure and composition, limiting the number of species with similar requirements that are capable to coexist [2]. In this study, the competitive pressure exerted by the ocelot on the southern tiger cat seems to affect the pattern of habitat use by the latter, reducing its occurrence in areas with higher ocelot occupancy probability.

Despite not having sufficient data to analyze the spatial interactions between ocelots and margays and jaguarundis, these last two species may also have mechanisms to avoid ocelot competition similar to those shown by the southern tiger cat. Since the jaguarundi has a daily activity pattern quite different from that of the ocelot, it may tolerate a greater overlap in habitat use with ocelots than the southern tiger cat and the margay. The margay presents high overlap of its daily activity pattern with the ocelot, so it is more likely to spatially avoid its presence, for example through differential use of the arboreal stratum, or making greater use of sites with low ocelot presence [18], but these hypotheses must still be tested.

Our results suggest that the landscape heterogeneity produced by human activities impacted the coexistence of these species, by generating habitats with different quality for them, spatially affecting their population’s status. Conserving large areas of continuous and well protected native forest is a requirement for the conservation of ocelots and other human-sensitive species like jaguars and pumas [39, 53]. Maintaining fragments of native forest immersed in human used matrices may ameliorate the negative effects of agricultural and timber production lands and favor the presence of the small felids. Further studies are needed to understand the characteristics of the forest fragments necessary to support viable populations of small felids, such as the minimum area and the degree of connectivity. Due to their interspecific relationships and their differential response to human impacts, conservation of medium and small felids in the Atlantic Forest clearly depends not only on conventional conservation strategies (e.g. large protected areas) but also on the planning and management of human land uses.

## Supporting information

S1 Fig Occupancy probability for ocelots. Location of the camera-trap stations (N = 184) with low (0–0.33, yellow), intermediate (0.34–0.66, orange), and high (0.67–1.00, red) **occupancy** probability of ocelots. Triangles = stations located in continuous forest, circles = forest fragment stations, squares = pine plantations.
(DOCX)

S1 Table *In situ* vegetation measurements to characterize forest type, and understory and arboreal structure from each camera-trap station. Measurements were realized at 10-m distance from the camera trap, towards the four cardinal points.
(DOCX)

S2 Table Covariates and their hypotheses and predictions. Covariates used to model the occupancy probability and detection probability for the four felids in the Single-species occupancy models and their respective hypotheses and predictions.
(DOCX)

S3 Table Expected changes in the daily activity patterns of the four felids due to human impact and for the three small felids due to the ocelot occurrence. Human impact was measured with the human cost of access and the ocelot occurrence with the occupancy probability estimated through the occupancy models.
(DOCX)

S4 Table Single-species single-season models for ocelot to estimate ψ (occupancy probability) and p (detection probability). We run the combination of all the covariates (N = 64) using *unmarked* package (Fiske and Chandler 2011) with the open-source software R 3.1.22 (R Core Team, 2014). Models were ordered according to the lowest value of AICc.
(DOCX)

S5 Table Beta estimates, their confidence intervals (CI 95%), and cumulative AICc weight for each covariate included in the set of best models for ocelots. Models were selected using the ΔAIC ≤2 for criteria.
(DOCX)

S6 Table Single-species single-season models for southern tiger cat to estimate ψ (occupancy probability) and p (detection probability). We run the combination of all the covariates (N = 64) using *unmarked* package (Fiske and Chandler 2011) with the open-source software R 3.1.22 (R Core Team, 2014). Models were ordered according to the lowest value of AICc.
(DOCX)

S7 Table Beta estimates, their confidence intervals (CI 95%), and cumulative AICc weight for each covariate included in the set of best models for southern tiger cats. Models were selected using the ΔAIC ≤2 for criteria.
(DOCX)

S8 Table Co-ocurrence models for ocelot (species A) and for southern tiger cat (species B). Models were ordered according to the lowest value of AICc. Models were run at PRESENCE 6.2.
(DOCX)

S9 Table Beta estimates and their confidence intervals (CI 95%) for each parameter included in the best model of the co-occurrence models for ocelots and southern tiger cats. (DOCX)

S10 Table Mean occupancy probability for southern tiger cats for the entire study area and discriminated by landscape condition (CF = continuous forest, FF = fragmented forest, PP = pine plantations). (DOCX)

S11 Table Frequency of stations with low (0–0.33), intermediate (0.34–0.66) and high (0.66–1) occupancy probability of southern tiger cat according to the landscape condition. (DOCX)

## References

[1] Jaksic F, Marone L. Ecología de comunidades, Segunda edición ampliada, Ediciones Universidad Católica de Chile. Santiago. 2007.

[2] Begon M, Harper JI, Colin CR. Ecología: individuos, poblaciones y comunidades Editorial Omega SA, Barcelona, España Diaphorina citri 1988.

[3] Schoener TW. Resource partitioning in ecological communities. science. 1974;185(4145):27–39 10.1126/science.185.4145.27 17779277

[4] Polis GA, Myers CA, Holt RD. The ecology and evolution of intraguild predation: potential competitors that eat each other. Annual review of ecology and systematics. 1989;20(1):297–330

[5] Tannerfeldt M, Elmhagen B, Angerbjorn A. Exclusion by interference competition? The relationship between red and arctic foxes. Oecologia. 2002;132:213–20. 10.1007/s00442-002-0967-8 . 28547354

[6] Steinmetz R, Seuaturien N, Chutipong W. Tigers, leopards, and dholes in a half-empty forest: Assessing species interactions in a guild of threatened carnivores. Biological Conservation. 2013;163:68–78. 10.1016/j.biocon.2012.12.016

[7] Di Bitetti M, Di Blanco Y, Pereira J, Paviolo A, Jiménez Perez I. Time partitioning favors the coexistence of sympatric crab-eating fox (*Cerdocyon thous*) and pampas fox (*Lycalopex gymnocercus*). Journal of Mammalogy. 2009;90(2):479–90.

[8] Palomares F, Caro TM. Interspecific killing among mammalian carnivores. The American Naturalist. 1999;153(5):492–508 10.1086/303189 29578790

[9] Donadio E, Buskirk SW. Diet, morphology, and interspecific killing in carnivora. American Naturalist. 2006;167:524–36. 10.1086/501033 . 16670995

[10] Kiltie RA. Size ratios among sympatric neotropical cats. Oecologia. 1984;61:411–6. 10.1007/BF00379644 . 28311072

[11] Bininda Emonds ORP, Gittleman JL, Purvis A. Building large trees by combining phylogenetic information: A complete phylogeny of the extant Carnivora (Mammalia). Biological Reviews of the Cambridge Philosophical Society. 1999;74:143–75. . 10396181 10.1017/s0006323199005307

[12] Sunquist M, Sunquist F. Wild cats of the world. Chicago, Illinois, USA: University of Chicago Press; 2002. 452 p.

[13] Johnson WE, Eizirik E, Pecon-Slattery J, Murphy WJ, Antunes A, Teeling E, et al The late miocene radiation of modern felidae: A genetic assesstment. Science. 2006;311:73–7. 10.1126/science.1122277 . 16400146

[14] Macdonald D, Loveridge A. The biology and conservation of wild felids: Oxford University Press; 2010.

[15] Wang E. Diets of ocelots (Leopardus pardalis), margays (L. wiedii), and oncillas (L. tigrinus) in the Atlantic Rainforest in southeast Brazil. Studies on Neotropical Fauna and Environment. 2002;37:207–12.

[16] Rocha-Mendes F, Mikich SB, Quadros J, Pedro WA. Feeding ecology of carnivores (Mammalia, Carnivora) in Atlantic forest remnants, southern Brazil. Biota Neotropica. 2010;10(4):21–30

[17] Rinaldi AR, Rodriguez FH, Carvalho AL, Passos FC. Feeding of small Neotropical felids (Felidae: Carnivora) and trophic niche overlap in antropized mosaic landscape, South Brazilian. Biotemas. 2015;28(4):155 10.5007/2175-7925.2015v28n4p155

[18] Di Bitetti M, De Angelo C, Di Blanco Y, Paviolo A. Niche partitioning and species coexistence in a Neotropical felid assemblage. Acta Oecologica. 2010;36:403–12.

[19] Oliveira-Santos LGR, Graipel ME, Tortato MA, Zucco CA, Cáceres NC, Goulart FVB. Abundance changes and activity flexibility of the oncilla, Leopardus tigrinus (Carnivora: Felidae), appear to reflect avoidance of conflict. Zoologia (Curitiba). 2012 10.1590/s1984-46702012000200003

[20] Massara RL, Paschoal AMO, Bailey LL, Doherty PF, Chiarello AG. Ecological interactions between ocelots and sympatric mesocarnivores in protected areas of the Atlantic Forest, southeastern Brazil. Journal of Mammalogy. 2016;97(6):1634–44. 10.1093/jmammal/gyw129

[21] de Oliveira TG, Tortato MA, Silveira L, Kasper CB, Mazim FD, Lucherini M, et al Ocelot ecology and its effect on the small-felid guild in the lowland neotropics. Biology and conservation of wild felids. 2010:559–80.

[22] de Oliveira TG, Pereira JA. Intraguild Predation and Interspecific Killing as Structuring Forces of Carnivoran Communities in South America. Journal of Mammalian Evolution. 2013;21(4):427–36. 10.1007/s10914-013-9251-4

[23] Nagy-Reis MB, Nichols JD, Chiarello AG, Ribeiro MC, Setz EZ. Landscape Use and Co-Occurrence Patterns of Neotropical Spotted Cats. PloS one. 2017;12(1):e0168441 10.1371/journal.pone.0168441 28052073 PMC5215768

[24] MacKenzie DI, Nichols JD, Royle JA, Pollock KH, Bailey LL, Hines JE. Occupancy estimation and modeling: inferring patterns and dynamics of species occurrence San Diego, USA: Academic Press; 2006. 344 p.

[25] Richmond OMW, Hines JE, Beissinger SR. Two‐species occupancy models: a new parameterization applied to co‐occurrence of secretive rails. Ecological Applications. 2010;20(7):2036–46. 21049888 10.1890/09-0470.1

[26] Crooks KR, Soule ME. Mesopredator release and avifaunal extinctions in a fragmented system. Nature. 1999;400(6744):563–6.

[27] Lewis JS, Bailey LL, VandeWoude S, Crooks KR. Interspecific interactions between wild felids vary across scales and levels of urbanization. Ecol Evol. 2015;5(24):5946–61. 10.1002/ece3.1812 26811767 PMC4717346

[28] Izquierdo A, De Angelo C, Aide M. Thirty years of human demography and land-use change in the Atlantic Forest of Misiones, Argentina: a test of the forest transition model. Ecology and Society. 2008;13(2):3.

[29] Giraudo AR, Abramson RR. Diversidad cultural y usos de la fauna silvestre por lo pobladores de la Selva Misionera ¿Una alternativa de conservación? In: Bertonatti C, Corcuera J, editors. La Situación Ambiental Argentina 2000. Buenos Aires, Argentina: Fundación Vida Silvestre Argentina; 2000 p. 233–43.

[30] Agostini I, Holzmann I, Di Bitetti MS. Are howler monkey species ecologically equivalent? Trophic niche overlap in syntopic *Alouatta guariba clamitans* and *Alouatta caraya*. American Journal of Primatology. 2010;72(2):173–86. 10.1002/ajp.20775 19953557

[31] Galindo-Leal C, de Gusmão Câmara I. Atlantic Forest Hotspot Status: An Overview In: Galindo-Leal C, de Gusmão Câmara I, editors. Atlantic Forest of South America: Biodiversity Status, Threats, and Outlook. Washington: Island Press; 2003 p. 3–11.

[32] Trigo TC, Tirelli FP, Machado LF, Peters FB, Indrusiak CB, Mazim FD, et al Geographic distribution and food habits ofLeopardus tigrinusandL. geoffroyi(Carnivora, Felidae) at their geographic contact zone in southern Brazil. Studies on Neotropical Fauna and Environment. 2013;48(1):56–67. 10.1080/01650521.2013.774789

[33] Myers N, Mittermeier RA, Mittermeier CG, da Fonseca GAB, Kent J. Biodiversity hotspots for conservation priorities. Nature. 2000;403:853–8. 10.1038/35002501 10706275

[34] Ribeiro MC, Metzger JP, Martensen AC, Ponzoni FJ, Hirota MM. The Brazilian Atlantic Forest: How much is left, and how is the remaining forest distributed? Implications for conservation. Biological Conservation. 2009;142(6):1141–53.

[35] Trentini CP, Campanello PI, Villagra M, Ritter L, Ares A, Goldstein G. Thinning of loblolly pine plantations in subtropical Argentina: Impact on microclimate and understory vegetation. Forest Ecology and Management. 2017;384:236–47

[36] Mueller-Dombois D, Ellenberg H. Aims and methods of vegetation ecology. 1974.

[37] Krebs CJ. Ecological methodology. Harper & Row New York; 1989.

[38] Di Bitetti M, Paviolo A, De Angelo C, Di Blanco Y. Local and continental correlates of the abundance of a neotropical cat, the ocelot (Leopardus pardalis). Journal of Tropical Ecology. 2008;24(2):189–200.

[39] Paviolo A, De Angelo C, Di Blanco Y, Di Bitetti M. Jaguar population decline in the Upper Paraná Atlantic Forest of Argentina and Brazil. Oryx. 2008;42(4):554–61. 10.1017/S0030605308000641

[40] Paviolo A, Di Blanco YE, De Angelo CD, Di Bitetti MS. Protection affects the abundance and activity patterns of pumas in the atlantic forest. Journal of Mammalogy. 2009;90(4):926–34.

[41] Paviolo A, De Angelo C, Ferraz KM, Morato RG, Pardo JM, Srbek-Araujo AC, et al A biodiversity hotspot losing its top predator: The challenge of jaguar conservation in the Atlantic Forest of South America. Scientific Reports. 2016;6. 10.1038/srep37147 PMC5111070 27849006

[42] Di Bitetti MS, Paviolo A, De Angelo C. Density, habitat use and activity patterns of ocelots (Leopardus pardalis) in the Atlantic Forest of Misiones, Argentina. Journal of Zoology. 2006;0(0). 10.1111/j.1469-7998.2006.00102.x

[43] MacKenzie DI, Nichols JD, Lachman GB, Droege S, Andrew Royle J, Langtimm CA. Estimating site occupancy rates when detection probabilities are less than one. Ecology. 2002;83(8):2248–55

[44] MacKenzie DI, Bailey LL, Nichols J. Investigating species co‐occurrence patterns when species are detected imperfectly. Journal of Animal Ecology. 2004;73(3):546–55%@ 1365–2656.

[45] Efford MG, Dawson DK. Occupancy in continuous habitat. Ecosphere. 2012;3(4):1–15

[46] Neilson EW, Avgar T, Burton AC, Broadley K, Boutin S. Animal movement affects interpretation of occupancy models from camera‐trap surveys of unmarked animals. Ecosphere. 2018;9(1).

[47] Di Rienzo JA. InfoStat versión 2009 Grupo InfoStat, FCA, Universidad Nacional de Córdoba, Argentina http://www.infostat.com.ar. 2009.

[48] Crawshaw PG Jr. Comparative ecology of ocelot Felis pardalis and jaguar Panthera onca in a protected subtropical forest in Brazil and Argentina [Dissertation] Gainesville, USA: University of Florida; 1995.

[49] Kasper CB, Schneider A, Oliveira TG. Home range and density of three sympatric felids in the Southern Atlantic Forest, Brazil. Braz J Biol. 2016;76(1):228–32. S1519-69842016005102106. 10.1590/1519-6984.19414 26871745

[50] Schadt S, Revilla E, Wiegand T, Knauer F, Kaczensky P, Breitenmoser U, et al Assessing the suitability of central European landscapes for the reintroduction of Eurasian lynx. Journal of Applied Ecology. 2002;39:189–203.

[51] Naves J, Revilla E, Delibes M, Wiegand T. Endangered species constrained by natural and human factors: the case of brown bears in Northern Spain. Conservation Biology. 2003;17:1276–89.

[52] Kanagaraj R, Wiegand T, Kramer-Schadt S, Anwar M, Goyal SP. Assessing habitat suitability for tiger in the fragmented Terai Arc Landscape of India and Nepal. Ecography. 2011;34(6):970–81. 10.1111/j.1600-0587.2010.06482.x

[53] De Angelo C, Paviolo A, Di Bitetti M. Differential impact of landscape transformation on pumas (*Puma concolor*) and jaguars (*Panthera onca*) in the Upper Paraná Atlantic Forest. Diversity and Distributions. 2011;17(3):422–36. 10.1111/j.1472-4642.2011.00746.x.

[54] De Angelo C, Paviolo A, Wiegand T, Kanagaraj R, Di Bitetti MS. Understanding species persistence for defining conservation actions: A management landscape for jaguars in the Atlantic Forest. Biological Conservation. 2013;159:422–33.

[55] Cruz P. Distribución, requerimientos de hábitat e interacciones ecológicas de los felinos medianos y pequeños del Bosque Atlántico del Alto Paraná de la provincia de Misiones [Ph.D. dissertation. ]. Buenos Aires University, Argentina.2017.

[56] Burnham KP, Anderson DR. Model selection and multimodel inference: a practical information-theoretic approach New York, USA: Springer-Verlag; 2002. 496 p.

[57] Anderson DR. Model based inference in the life sciences: a primer on evidence: Springer Science & Business Media; 2007.

[58] Doherty PF, White GC, Burnham KP. Comparison of model building and selection strategies. Journal of Ornithology. 2012;152(2):317–23

[59] Fiske I, Chandler R. Unmarked: an R package for fitting hierarchical models of wildlife occurrence and abundance. Journal of Statistical Software. 2011;43(10):1–23.

[60] R Development Core Team. R: A language and environment for statistical computing. Vienna, Austria: R Foundation for Statistical Computing; 2012.

[61] MacKenzie DI, Bailey LL. Assessing the fit of site-occupancy models. Journal of Agricultural, Biological, and Environmental Statistics. 2004;9(3):300–18.

[62] Dugger KM, Forsman ED, Franklin AB, Davis RJ, White GC, Schwarz CJ, et al The effects of habitat, climate, and Barred Owls on long-term demography of Northern Spotted Owls. The Condor. 2015;118(1):57–116.

[63] Fisher RA. The logic of inductive inference. Journal of the Royal Statistical Society. 1935;98(1):39–82.

[64] Hines JE. PRESENCE Software to estimate patch occupancy and related parameters. http://www.mbr-pwrc.usgs.gov/software/presence.html. 2006.

[65] Farris ZJ, Karpanty SM, Ratelolahy F, Kelly MJ. Predator–Primate Distribution, Activity, and Co-occurrence in Relation to Habitat and Human Activity Across Fragmented and Contiguous Forests in Northeastern Madagascar. International Journal of Primatology. 2014;35(5):859–80. 10.1007/s10764-014-9786-0

[66] Farris ZJ, Kelly MJ, Karpanty S, Ratelolahy F. Patterns of spatial co‐occurrence among native and exotic carnivores in north‐eastern Madagascar. Animal Conservation. 2016;19(2):189–98

[67] Steen DA, McClure CJW, Brock JC, Craig Rudolph D, Pierce JB, Lee JR, et al Snake co‐occurrence patterns are best explained by habitat and hypothesized effects of interspecific interactions. Journal of Animal Ecology. 2014;83(1):286–95. 10.1111/1365-2656.12121 23998642

[68] Worton BJ. Kernel methods for estimating the utilization distribution in home-range studies. Ecology. 1989;70(1):164–8.

[69] Meredith M, Ridout M. overlap: Estimates of coefficient of overlapping for animal activity patterns. R package version 02. 2014;4.

[70] Oliveira-Santos LGR, Zucco CA, Agostinelli C. Using conditional circular kernel density functions to test hypotheses on animal circadian activity. Animal Behaviour. 2013;85(1):269–80. 10.1016/j.anbehav.2012.09.033

[71] Ridout MS, Linkie M. Estimating overlap of daily activity patterns from camera trap data. Journal of Agricultural, Biological, and Environmental Statistics. 2009;14(3):322–37.

[72] Batschelet E. Circular statistics in biology Academic Press, New York 1981.

[73] Emmons L. A field study of ocelots (Felis pardalis) in Peru. Revue D Ecologie–La Terre Et La Vie. 1988;43: 133–157.

[74] Haines AM, Tewes ME, Laack LL, Horne JS, Young JH. A habitat-based population viability analysis for ocelots (Leopardus pardalis) in the United States. Biological Conservation. 2006;132:424–36.

[75] Massara RL, Paschoal AM, Doherty PF Jr., Hirsch A, Chiarello AG. Ocelot Population Status in Protected Brazilian Atlantic Forest. PloS one. 2015;10(11):e0141333 10.1371/journal.pone.0141333 26560347 PMC4641647

[76] Whiteman CW, Matushima ER, Cavalcanti Confalonieri UE, Palha MdDC, da Silva AdSL, Monteiro VC. Human and domestic animal populations as a potential threat to wild carnivore conservation in a fragmented landscape from the Eastern Brazilian Amazon. Biological Conservation. 2007;138(1–2):290–6.

[77] de Almeida Curi NH, de Oliveira Paschoal AM, Massara RL, Marcelino AP, Ribeiro AA, Passamani M, et al Factors associated with the seroprevalence of leishmaniasis in dogs living around Atlantic Forest fragments. PloS one. 2014;9(8):e104003 10.1371/journal.pone.0104003 25089629 PMC4121198

[78] Massara R, Paschoal AMdO, L. Bailey L, F. Doherty P Jr, Hirsch A, G. Chiarello A. Factors influencing ocelot occupancy in Brazilian Atlantic Forest reserves. Biotropica. 2018;50(1):125–34.

[79] Hill K, Padwe J, Bejyvagi C, Bepurangi A, Jakugi F, Tykuarangi R, et al Impact of hunting on large vertebrates in the Mbaracayu Reserve, Paraguay. Conservation Biology. 1997;11(6):1339–53

[80] Caro T. Densities of mammals in partially protected areas: The Katavi ecosystem of Western Tanzania. Journal of Applied Ecology. 1999;36(2):205–17.

[81] Peres CA, Lake IR. Extent of nontimber resource extraction in tropical forests: Accessibility to game vertebrates by hunters in the Amazon Basin. Conservation Biology. 2003;17(2):521–35.

[82] Laurance WF, Croes BM, Tchignoumba L, Lahm SA, Alonso A, Lee ME, et al Impacts of roads and hunting on central African rainforest mammals. Conservation Biology. 2006;20(4):1251–61 16922241 10.1111/j.1523-1739.2006.00420.x

[83] Di Bitetti MS, Paviolo A, Ferrari C, De Angelo C, Di Blanco Y. Differential responses to hunting in two sympatric species of brocket deer (*Mazama americana* and *Mazama nana*). Biotropica. 2008;40(5):636–45.

[84] Paviolo A. Densidad de yaguareté (*Panthera onca*) en la Selva Paranaense: su relación con la abundancia de presas, presión de caza y coexistencia con el puma (Puma concolor) Córdoba, Argentina: Universidad Nacional de Córdoba; 2010.

[85] Michalski F, Peres CA. Anthropogenic determinants of primate and carnivore local extinctions in a fragmented forest landscape of southern Amazonia. Biological Conservation. 2005;124:383–96.

[86] Harmsen B, Foster R, Silver S, Ostro L, Doncaster C. Differential use of trails by forest mammals and the implications for camera-trap studies: A case study from Belize. Biotropica. 2010;42(1):126–33.

[87] Di Bitetti MS, Paviolo A, De Angelo C. Camera trap photographic rates on roads vs. off roads: location does matter. Mastozoología neotropical. 2014;21(1):37–46

[88] Emmons L, Feer F. Neotropical rainforest mammals: a field guide1997.

[89] de Oliveira TG. Leopardus wiedii. Mammalian Species. 1998;(579):1–6

[90] Morato RG, Stabach JA, Fleming CH, Calabrese JM, De Paula RC, Ferraz KM, et al Space Use and Movement of a Neotropical Top Predator: The Endangered Jaguar. PloS one. 2016;11(12):e0168176 10.1371/journal.pone.0168176 28030568 PMC5193337

[91] Moreira-Arce D, Vergara PM, Boutin S, Simonetti JA, Briceño C, Acosta-Jamett G. Native forest replacement by exotic plantations triggers changes in prey selection of mesocarnivores. Biological Conservation. 2015;192:258–67

[92] Kasper CB, Mazim F, Soares JBG, de Oliveira TG, Fabián ME. Composição e abundância relativa dos mamíferos de médio e grande porte no Parque Estadual do Turvo, Rio Grande do Sul, Brasil. Revista Brasileira de Zoología. 2007;24(4):1087–100.

[93] de Oliveira TG. Herpailurus yagouaroundi. Mammalian Species. 1998;578:1–6.

[94] Maffei L, Noss A, Fiorello C. The jaguarundi (Puma yagouaroundi) in the kaa-iya del gran Chaco National Park, Santa Cruz, Bolivia. Mastozoología neotropical. 2007;14(2):263–6.

[95] Vanderhoff EN, Hodge A-M, Arbogast BS, Nilsson J, Knowles TW. Abundance and activity patterns of the margay (Leopardus wiedii) at a mid-elevation site in the eastern Andes of Ecuador. Mastozoología neotropical. 2011;18(2):271–9.

[96] Briones-Salas M, Lira-Torres I, Carrera-Treviño R, Sánchez-Rojas G. Relative abundance and activity patterns of wild felids in Chimalapas rainforest, Oaxaca, Mexico. Therya. 2016;7(1):123–34. 10.12933/therya-16-320

[97] Pérez-Irineo G, Santos-Moreno A. Abundance and activity patterns of medium-sized felids (Felidae, Carnivora) In Southeastern Mexico. The Southwestern Naturalist. 2016;61(1):33–9. 10.1894/0038-4909-61.1.33

[98] Massara RL, de Oliveira Paschoal AM, Bailey LL, Doherty PF Jr, de Frias Barreto M, Chiarello AG. Effect of humans and pumas on the temporal activity of ocelots in protected areas of Atlantic Forest. Mammalian Biology. 2018;92:86–93.

