# Supplementary material for: Effects of human impacts on habitat use, activity patterns and ecological relationships among medium and small felids of the Atlantic Forest
Source: PLoS One. 2018 Aug 1;13(8):e0200806. doi: 10.1371/journal.pone.0200806 (PMC6070200; doi:10.1371/journal.pone.0200806)
Supplement: S1 Fig — Location of the camera-trap stations (N = 184) with low (0–0.33, yellow), intermediate (0.34–0.66, orange), and high (0.67–1.00, red) occupancy probability of ocelots. Triangles = stations located in continuous forest, circles = forest fragment stations, squares = pine plantations. (DOCX) [file pone.0200806.s001.docx]

S1 Fig. Probability of occurrence for ocelots. Location of the camera-trap stations (N=184) with low (0 – 0.33, yellow), intermediate (0.34 – 0.66, orange), and high (0.67 – 1.00, red) probability of occurrence of ocelots. Triangles = stations located in continuous forest, circles = forest fragment stations, squares = pine plantations.
