# Supplementary material for: Effects of human impacts on habitat use, activity patterns and ecological relationships among medium and small felids of the Atlantic Forest
Source: PLoS One. 2018 Aug 1;13(8):e0200806. doi: 10.1371/journal.pone.0200806 (PMC6070200; doi:10.1371/journal.pone.0200806)
Supplement: S1 Table — Measurements were realized at 10-m distance from the camera trap, towards the four cardinal points. (DOCX) [file pone.0200806.s002.docx]

S1 Table. ***In situ* vegetation measurements to characterize forest type, and understory and arboreal structure from each camera-trap station.** Measurements were realized at 10-m distance from the camera trap, towards the four cardinal points.

| Measurement | Description |
| --- | --- |
| Relative abundance of dominant species or genera of the understory | We used a relative scale with values from 0 (absence) to 3 (maximum possible abundance for each species) to define the relative abundance of: *Sorocea bonplandii*, *Piper* sp., *Chusquea ramosissima,* *Guadua trinii*, *Merostachis clausenii*, and other vegetation forms, lianas, ferns, grasses, considered characteristic of different types of forest environments in Misiones (primary forests in good conservation status, highly intervened forests and secondary forests). |
| Understory density | We used the punctual interception method (Mueller-Dombois and Ellenberg 1974). We counted the number of times the vegetation contacted each of the four 1-m segments of a 4-m tall rod positioned vertically at each of the four points located 10 m along the transect from the location point of the camera. |
| Tree density and basal area | We use the point-centered quarter method (Krebs 1989). At each of the four points we divided the terrain into four quadrants and measured the distance and diameter of the nearest tree (with a DBH> 10 cm) from each quadrant. |
