# Supplementary material for: Effects of human impacts on habitat use, activity patterns and ecological relationships among medium and small felids of the Atlantic Forest
Source: PLoS One. 2018 Aug 1;13(8):e0200806. doi: 10.1371/journal.pone.0200806 (PMC6070200; doi:10.1371/journal.pone.0200806)
Supplement: S2 Table — Covariates used to model the occupancy probability and detection probability for the four felids in the Single-species occupancy models and their respective hypotheses and predictions. (DOCX) [file pone.0200806.s003.docx]

S2 Table. **Covariates and their hypotheses and predictions**. Covariates used to model the probability of occurrence and detection probability for the four felids in the single-species occupancy models and their respective hypotheses and predictions.

| **Covariates** | **Hypotheses and Predictions** |
| --- | --- |
| **Landscape condition**. It refers to three different environments with different degrees of human intervention: continuous forest areas, mainly protected areas (CF), forest fragments and strips immersed in a pine plantations matrix or in a rural matrix (FF), and pine (*Pinus taeda*) plantations (PP). | H: The probability of occurrence of the four felids is negatively affected by the degree of anthropic intervention, being the ocelot more sensitive than the small felids to the highest degree of environmental modification.  P: The probability of occurrence of the four felids will be higher in sites with continuous forest and lower in sites with pine plantations. We also expect that ocelots will be more affected than the small cats. |
| **Cost of human access**: is the estimated time it would take to a human being to access each sampling site from the nearest urban location | H: The probability of occurrence of the four felids is negatively affected by the presence of people.  P: The probability of occurrence of the four felids will increase with the human cost of access. |
| **Vegetation structure:** Values of axis 1 of a Principal Component Analysis base on the *in situ* vegetation measurements (S1 Table). Positive values represent sites with higher structural complexity (native forest sites with higher diversity of strata and species), and negative values indicating sites with scant understory vegetation (pine plantations) | H: The probability of occurrence of the four felids will be higher in the original, more pristine native forest and high diversity of strata and vegetable species that serve as cover for foraging and protection.  P: The probability of occurrence of the four felids will be higher in sites with more complex vegetation structure. |
| **Percentage of native forest in a 2-km radius**: the abundance of forest in the area around each camera trap location | H: The probability of occurrence of the four felids will be favored in sites surrounded by their preferred habitat type: continuous native forests.  P: The probability of occurrence of the four felids will be higher in sites with higher percentage of native forest. |
| **Prey recording rate**: recording rate (records/100 days) of the potential main prey for each felid. | H: The abundance (and thus the probability of detection and occurrence) of a carnivore species increases with prey availability.  P: The probability of occurrence of the four felids will be higher in sites with higher prey recording rates. |
| **Contact points of the vegetation on a 1-m high rod**: number of times the vegetation contacted the first 1-m segment of a 4-m tall rod positioned vertically at each of the four points located 10 m along the transect from the location point of the camera. | H: The vegetation of the understory may reduce or obstruct the effectively surveyed area of the camera traps and/or the identification of the species if the camera is triggered, thus missing potential records.  P: The detection probability of the four felids will decrease with the contact points of the vegetation. |
