# Supplementary material for: Effects of human impacts on habitat use, activity patterns and ecological relationships among medium and small felids of the Atlantic Forest
Source: PLoS One. 2018 Aug 1;13(8):e0200806. doi: 10.1371/journal.pone.0200806 (PMC6070200; doi:10.1371/journal.pone.0200806)
Supplement: S3 Table — Human impact was measured with the human cost of access and the ocelot occurrence with the occupancy probability estimated through the occupancy models. (DOCX) [file pone.0200806.s004.docx]

S3 Table. **Expected changes in the daily activity patterns of the four felids due to human impact and for the three small felids due to the ocelot occurrence.** Human impact was measured as the human cost of access and the probability of ocelot occurrence was estimated with occupancy models.

| **Species** | **Human impact** | **Ocelot occurrence** |
| --- | --- | --- |
| **Ocelot** | Avoids crepuscular hours and becomes more nocturnal | - |
| **Jaguarundi** | Becomes more crepuscular | Avoids crepuscular hours when ocelots may still be active |
| **Margay** | No response since humans are active during daylight hours | No response or may avoid peak activity of ocelots |
| **S. tiger cat** | Since it is a cathemeral species it becomes more nocturnal | Since it is a cathemeral species it becomes more diurnal |
