# Supplementary material for: Effects of human impacts on habitat use, activity patterns and ecological relationships among medium and small felids of the Atlantic Forest
Source: PLoS One. 2018 Aug 1;13(8):e0200806. doi: 10.1371/journal.pone.0200806 (PMC6070200; doi:10.1371/journal.pone.0200806)
Supplement: S4 Table — We run the combination of all the covariates (N = 64) using unmarked package (Fiske and Chandler 2011) with the open-source software R 3.1.22 (R Core Team, 2014). Models were ordered according to the lowest value of AICc. (DOCX) [file pone.0200806.s005.docx]

S4 Table**. Single-species single-season models for ocelot to estimate ψ (probability of occurrence) and p (detection probability).** We run the combination of all the covariates (N=64) using *unmarked* package (Fiske and Chandler 2011) with the open-source software R 3.1.22 (R Core Team, 2014). Models were ordered according to the lowest value of AICc.

| Models | nPars | AICc | ΔAICc | AICcwt |
| --- | --- | --- | --- | --- |
| ψ (Land+access)p(.) | 5 | 272.86 | 0 | 0.11 |
| ψ (Land +access)p(cont) | 6 | 273.5 | 0.64 | 0.08 |
| ψ (Land +access+forest)p(.) | 6 | 273.69 | 0.83 | 0.07 |
| ψ (Land)p(.) | 4 | 274.32 | 1.46 | 0.05 |
| ψ (Land +access+forest)p(cont) | 7 | 274.43 | 1.57 | 0.05 |
| ψ (Land +access+Veget)p(.) | 6 | 274.45 | 1.59 | 0.05 |
| ψ (access+Veget)p(.) | 4 | 274.81 | 1.95 | 0.04 |
| ψ (Land +access+prey)p(.) | 6 | 274.96 | 2.09 | 0.04 |
| ψ (Land)p(cont) | 5 | 275.05 | 2.19 | 0.04 |
| ψ (Land +access+Veget)p(cont) | 7 | 275.25 | 2.39 | 0.03 |
| ψ (access+Veget)p(cont) | 5 | 275.31 | 2.44 | 0.03 |
| ψ (Land +access+forest+Veg)p(.) | 7 | 275.36 | 2.49 | 0.03 |
| ψ (Land +access+prey)p(cont) | 7 | 275.6 | 2.74 | 0.03 |
| ψ (Land +access+forest+roed)p(.) | 7 | 275.78 | 2.92 | 0.02 |
| ψ (Land +Veget)p(.) | 5 | 275.83 | 2.97 | 0.02 |
| ψ (Land +forest)p(.) | 5 | 276.14 | 3.28 | 0.02 |
| ψ (Land +access+forest+Veget)p(cont) | 8 | 276.31 | 3.45 | 0.02 |
| ψ (Land +Veget)p(cont) | 6 | 276.42 | 3.56 | 0.02 |
| ψ (Land+access+Veget+prey)p(.) | 7 | 276.52 | 3.66 | 0.02 |
| ψ (Land +access+forest+prey)p(cont) | 8 | 276.53 | 3.66 | 0.02 |
| ψ (access+forest+Veget)p(.) | 5 | 276.77 | 3.9 | 0.02 |
| ψ (access+Veget+prey)p(.) | 5 | 276.88 | 4.01 | 0.01 |
| ψ (Land +prey)p(.) | 5 | 276.88 | 4.01 | 0.01 |
| ψ (Land +forest)p(cont) | 6 | 276.93 | 4.06 | 0.01 |
| ψ (Land +prey)p(cont) | 6 | 277.08 | 4.21 | 0.01 |
| ψ (Land +access+Veget+prey)p(cont) | 8 | 277.31 | 4.45 | 0.01 |
| ψ (access+forest+Veget)p(cont) | 6 | 277.34 | 4.47 | 0.01 |
| ψ (Land +access+forest+Veget+prey)p(.) | 8 | 277.4 | 4.54 | 0.01 |
| ψ (access+Veget+prey)p(cont) | 6 | 277.43 | 4.56 | 0.01 |
| ψ (Land +forest+Veget)p(.) | 6 | 277.62 | 4.76 | 0.01 |
| ψ (Land +Veget+prey)p(.) | 6 | 277.86 | 5 | 0.01 |
| ψ (access+forest)p(cont) | 5 | 277.99 | 5.12 | 0.01 |
| ψ (Land +forest+prey)p(.) | 6 | 278.18 | 5.32 | 0.01 |
| ψ (Land +forest+Veget)p(cont) | 7 | 278.32 | 5.46 | 0.01 |
| ψ (Land +Veget+prey)p(cont) | 7 | 278.35 | 5.48 | 0.01 |
| ψ (Land +access+forest+Veget+prey)p(cont) | 9 | 278.36 | 5.5 | 0.01 |
| ψ (access)p(cont) | 4 | 278.53 | 5.66 | 0.01 |
| ψ (access+forest)p(.) | 4 | 278.89 | 6.03 | 0.01 |
| ψ (access+forest+Veget+prey)p(.) | 6 | 278.9 | 6.04 | 0.01 |
| ψ (Land +forest+prey)p(cont) | 7 | 279 | 6.14 | 0 |
| ψ (access+forest+Veget+prey)p(cont) | 7 | 279.49 | 6.63 | 0 |
| ψ (Land +forest+Veget+prey)p(.) | 7 | 279.72 | 6.86 | 0 |
| ψ (access+prey)p(cont) | 5 | 279.86 | 7 | 0 |
| ψ (access+forest+prey)p(cont) | 6 | 280.11 | 7.25 | 0 |
| ψ (access+prey)p(.) | 4 | 280.13 | 7.27 | 0 |
| ψ (Land +forest+Veget+prey)p(cont) | 8 | 280.26 | 7.39 | 0 |
| ψ (access+forest+prey)p(.) | 5 | 280.65 | 7.79 | 0 |
| ψ (access)p(.) | 3 | 280.75 | 7.89 | 0 |
| ψ (forest+prey)p(.) | 4 | 282.65 | 9.79 | 0 |
| ψ (forest+prey)p(cont) | 5 | 282.96 | 10.09 | 0 |
| ψ (forest+Veget+prey)p(.) | 5 | 283.6 | 10.74 | 0 |
| ψ (forest+Veget+prey)p(cont) | 6 | 284.29 | 11.43 | 0 |
| ψ (Veget+prey)p(.) | 4 | 284.33 | 11.47 | 0 |
| ψ (forest+Veget)p(.) | 4 | 284.41 | 11.54 | 0 |
| ψ (prey)p(cont) | 4 | 284.46 | 11.6 | 0 |
| ψ (forest)p(.) | 3 | 284.76 | 11.89 | 0 |
| ψ (forest)p(cont) | 4 | 284.79 | 11.93 | 0 |
| ψ (Veget+prey)p(cont) | 5 | 284.91 | 12.05 | 0 |
| ψ (forest+Veget)p(cont) | 5 | 285.1 | 12.23 | 0 |
| ψ (prey)p(.) | 3 | 285.63 | 12.77 | 0 |
| ψ (Veget)p(.) | 3 | 289.34 | 16.48 | 0 |
| ψ (Veget)p(cont) | 4 | 290.47 | 17.61 | 0 |
| ψ (.)p(cont) | 3 | 298.37 | 25.51 | 0 |
| ψ (.)p(.) | 2 | 300.1 | 27.23 | 0 |

Land= landscape condition (continuous forest, fragmented forest, pine plantations), access= human cost of access, forest= percentage of native forest in a 2-km radius, Veget= vegetation structure (PCA axis 1), prey= recording rate of the potential main preys, cont= contacts point number of the understory vegetation in a 1-m rod.
