# Supplementary material for: Effects of human impacts on habitat use, activity patterns and ecological relationships among medium and small felids of the Atlantic Forest
Source: PLoS One. 2018 Aug 1;13(8):e0200806. doi: 10.1371/journal.pone.0200806 (PMC6070200; doi:10.1371/journal.pone.0200806)
Supplement: S5 Table — Models were selected using the ΔAIC ≤2 for criteria. (DOCX) [file pone.0200806.s006.docx]

S5 Table. **Beta estimates, their confidence intervals (CI 95%), and cumulative AICc weight for each covariate included in the set of best models for ocelots.** Models were selected using the ΔAICc ≤2 for criteria**.**

|  |  |  | Beta estimates (CI 95%) | | | | | | | | |
| --- | --- | --- | --- | --- | --- | --- | --- | --- | --- | --- | --- |
| ID | Models | ΔΑΙCc | Intercept ψ | Land CF | Land FF | access | forest | veget | prey | Intercept *p* | cont |
| 1 | ψ (Land+access)p(.) | 272.86 | -2.19 | 2.76 | 0.46 | 1.22 |  |  |  | -1.88 |  |
|  |  |  | (-3.33 to -1.05) | (1.17 to 4.35) | (-1.14 to 2.06) | (0.11 to 2.32) |  |  |  | (-2-30 to 1.47) |  |
| 2 | ψ (Land +access)p(cont) | 273.5 | -1.96 | 2.74 | 0.35 | 1.31 |  |  |  | -2.05 | 0.25 |
|  |  |  | (-3.22 to -0.71) | (1.03 to 4.45) | (-1.31 to 2.00) | (0.08 to 2.54) |  |  |  | (-2.53 to -1.58) | (-0.09 to 0.58) |
| 3 | ψ (Land +access+forest)p(.) | 273.69 | -2.71 | 4.18 | 0.78 | 1.58 | -0.8 |  |  | -1.90 |  |
|  |  |  | (-4.14 to -1.27) | (1.28 to 7.08) | (-0.93 to 2.48) | (0.21 to 2.94) | (-2.00 to 0.41) |  |  | (-2.30 to -1.50) |  |
| 4 | ψ (Land)p(.) | 274.32 | -2.25 | 3.21 | -0.03 |  |  |  |  | -1.84 |  |
|  |  |  | (-3.33 to -1.17) | (1.55 to 4.87) | (-1.48 to 1.42) |  |  |  |  | (-2.36 to -1.32) |  |
| 5 | ψ (Land +access+forest)p(cont) | 274.43 | -2.49 | 4.11 | 0.63 | 1.65 | -0.77 |  |  | -2.05 | 0.23 |
|  |  |  | (-4.01 to -0.98) | (1.21 to 7.01) | (-1.11 to 2.36) | (0.22 to 3.08) | (-1.97 to 0.44) |  |  | (-2.50 to -1.60) | (-0.09 to 0.56) |
| 6 | ψ (Land +access+Veget)p(.) | 274.45 | -1.63 | 1.79 | -0.34 | 1.21 |  | 0.54 |  | -1.90 |  |
|  |  |  | (-3.34 to 0.08) | (-0.88 to 4.46) | (-2.81 to 2.14) | (0.11 to 2.31) |  | (-0.72 to 1.80) |  | (-2.31 to -1.48) |  |
| 7 | ψ (access+Veget)p(.) | 274.81 | -0.94 |  |  | 2.04 |  | 1.19 |  | -2.01 |  |
|  |  |  | (-1.91 to 0.02) |  |  | (0.52 to 3.55) |  | (0.32 to 2.05) |  | (-2.48 to -1.53) |  |
| cumulative AICc weight | | |  | 0.82 | 0.82 | 0.77 | 0.36 | 0.41 | 0.26 |  | 0.42 |
