# Supplementary material for: Effects of human impacts on habitat use, activity patterns and ecological relationships among medium and small felids of the Atlantic Forest
Source: PLoS One. 2018 Aug 1;13(8):e0200806. doi: 10.1371/journal.pone.0200806 (PMC6070200; doi:10.1371/journal.pone.0200806)
Supplement: S6 Table — We run the combination of all the covariates (N = 64) using unmarked package (Fiske and Chandler 2011) with the open-source software R 3.1.22 (R Core Team, 2014). Models were ordered according to the lowest value of AICc. (DOCX) [file pone.0200806.s007.docx]

S6 Table. **Single-species single-season models for southern tiger cat to estimate ψ (probability of occurrence) and p (detection probability).** We run the combination of all the covariates (N=64) using *unmarked* package (Fiske and Chandler 2011) with the open-source software R 3.1.22 (R Core Team, 2014). Models were ordered according to the lowest value of AICc.

| Models | nPars | AICc | ΔAICc | AICcwt |
| --- | --- | --- | --- | --- |
| ψ (access)p(.) | 3 | 295.83 | 0 | 0.09 |
| ψ (access)p(cont) | 4 | 296.1 | 0.27 | 0.08 |
| ψ (.)p(.) | 2 | 296.28 | 0.45 | 0.07 |
| ψ (.)p(cont) | 3 | 296.99 | 1.16 | 0.05 |
| ψ (access+forest)p(.) | 4 | 297.63 | 1.8 | 0.04 |
| ψ (access+prey)p(.) | 4 | 297.65 | 1.81 | 0.04 |
| ψ (access+prey)p(cont) | 5 | 297.88 | 2.05 | 0.03 |
| ψ (access+Veget)p(.) | 4 | 297.91 | 2.08 | 0.03 |
| ψ (access+forest)p(cont) | 5 | 298.12 | 2.29 | 0.03 |
| ψ (access+Veget)p(cont) | 5 | 298.14 | 2.31 | 0.03 |
| ψ (forest)p(.) | 3 | 298.19 | 2.36 | 0.03 |
| ψ (Veget)p(.) | 3 | 298.25 | 2.42 | 0.03 |
| ψ (prey)p(.) | 3 | 298.34 | 2.51 | 0.02 |
| ψ (Veget)p(cont) | 4 | 298.55 | 2.72 | 0.02 |
| ψ (forest)p(cont) | 4 | 298.62 | 2.79 | 0.02 |
| ψ (Land)p(.) | 4 | 298.66 | 2.83 | 0.02 |
| ψ (prey)p(cont) | 4 | 299.07 | 3.24 | 0.02 |
| ψ (Land +access)p(.) | 5 | 299.31 | 3.47 | 0.02 |
| ψ (Land)p(cont) | 5 | 299.38 | 3.55 | 0.02 |
| ψ (access+forest+prey)p(.) | 5 | 299.51 | 3.68 | 0.02 |
| ψ (access+Veget+prey)p(cont) | 6 | 299.73 | 3.9 | 0.01 |
| ψ (access+forest+Veget)p(.) | 5 | 299.74 | 3.9 | 0.01 |
| ψ (access+Veget+prey)p(.) | 5 | 299.76 | 3.92 | 0.01 |
| ψ (Land +Veget)p(.) | 5 | 299.76 | 3.92 | 0.01 |
| ψ (access+forest+prey)p(cont) | 6 | 299.81 | 3.97 | 0.01 |
| ψ (Land +access)p(cont) | 6 | 299.95 | 4.12 | 0.01 |
| ψ (access+forest+Veget)p(cont) | 6 | 300.03 | 4.19 | 0.01 |
| ψ (Land +Veget)p(cont) | 6 | 300.09 | 4.26 | 0.01 |
| ψ (Veget+prey)p(cont) | 5 | 300.16 | 4.33 | 0.01 |
| ψ (forest+prey)p(.) | 4 | 300.26 | 4.43 | 0.01 |
| ψ (Veget+prey)p(.) | 4 | 300.28 | 4.44 | 0.01 |
| ψ (forest+Veget)p(cont) | 5 | 300.29 | 4.45 | 0.01 |
| ψ (Land +access+Veget)p(.) | 6 | 300.5 | 4.67 | 0.01 |
| ψ (forest+prey)p(cont) | 5 | 300.63 | 4.79 | 0.01 |
| ψ (Land +prey)p(.) | 5 | 300.64 | 4.8 | 0.01 |
| ψ (Land +forest)p(.) | 5 | 300.76 | 4.93 | 0.01 |
| ψ (Land +access+Veget)p(cont) | 7 | 301.07 | 5.24 | 0.01 |
| ψ (Land +prey)p(cont) | 6 | 301.12 | 5.28 | 0.01 |
| ψ (Land +access+forest)p(.) | 6 | 301.29 | 5.46 | 0.01 |
| ψ (Land +access+prey)p(.) | 6 | 301.31 | 5.48 | 0.01 |
| ψ (Land +Veget+prey)p(cont) | 7 | 301.45 | 5.62 | 0.01 |
| ψ (Land +Veget+prey)p(.) | 6 | 301.48 | 5.65 | 0.01 |
| ψ (Land +forest)p(cont) | 6 | 301.49 | 5.66 | 0.01 |
| ψ (access+forest+Veget+prey)p(cont) | 7 | 301.54 | 5.71 | 0.01 |
| ψ (access+forest+Veget+prey)p(.) | 6 | 301.7 | 5.87 | 0.01 |
| ψ (Land +access+prey)p(cont) | 7 | 301.86 | 6.02 | 0.01 |
| ψ (Land +access+forest)p(cont) | 7 | 301.92 | 6.09 | 0.01 |
| ψ (Land +forest+Veget)p(.) | 6 | 302.01 | 6.18 | 0.00 |
| ψ (Land +forest+Veget)p(cont) | 7 | 302.24 | 6.41 | 0.00 |
| ψ (forest+Veget+prey)p(cont) | 6 | 302.27 | 6.43 | 0.00 |
| ψ (Land +access+Veget+prey)p(cont) | 8 | 302.53 | 6.7 | 0.00 |
| ψ (Land +access+Veget+prey)p(.) | 7 | 302.63 | 6.79 | 0.00 |
| ψ (Land +access+forest+Veget)p(.) | 7 | 302.65 | 6.82 | 0.00 |
| ψ (Land +forest+prey)p(.) | 6 | 302.73 | 6.89 | 0.00 |
| ψ (Land +access+forest+Veget)p(cont) | 8 | 303.08 | 7.25 | 0.00 |
| ψ (Land +forest+prey)p(cont) | 7 | 303.18 | 7.35 | 0.00 |
| ψ (Land +access+forest+prey)p(.) | 7 | 303.2 | 7.37 | 0.00 |
| ψ (Land +forest+Veget+prey)p(cont) | 8 | 303.49 | 7.66 | 0.00 |
| ψ (Land +forest+Veget+prey)p(.) | 7 | 303.5 | 7.66 | 0.00 |
| ψ (Land +access+forest+prey)p(cont) | 8 | 303.61 | 7.77 | 0.00 |
| ψ (forest+Veget)p(.) | 4 | 303.72 | 7.88 | 0.00 |
| ψ (Land +access+forest+Veget+prey)p(cont) | 9 | 303.79 | 7.95 | 0.00 |
| ψ (forest+Veget+prey)p(.) | 5 | 304.39 | 8.55 | 0.00 |
| ψ (Land +access+forest+Veget+prey)p(.) | 8 | 304.47 | 8.64 | 0.00 |

Land= landscape condition (continuous forest, fragmented forest, pine plantations), access= human cost of access, forest= percentage of native forest in a 2-km radius, Veget= vegetation structure (PCA axis 1), prey= recording rate of the potential main preys, cont= contacts point number of the understory vegetation in a 1-m rod.
