# Supplementary material for: Effects of human impacts on habitat use, activity patterns and ecological relationships among medium and small felids of the Atlantic Forest
Source: PLoS One. 2018 Aug 1;13(8):e0200806. doi: 10.1371/journal.pone.0200806 (PMC6070200; doi:10.1371/journal.pone.0200806)
Supplement: S7 Table — Models were selected using the ΔAIC ≤2 for criteria. (DOCX) [file pone.0200806.s008.docx]

S7 Table. **Beta estimates, their confidence intervals (CI 95%), and cumulative AICc weight for each covariate included in the set of best models for southern tiger cats.** Models were selected using the ΔAICc ≤2 for criteria**.**

|  |  |  | Beta estimates (CI 95%) | | | | | |
| --- | --- | --- | --- | --- | --- | --- | --- | --- |
| ID | Models | ΔAICc | Intercept ψ | access | forest | prey | Intercept *p* | cont |
| 1 | ψ (access)p(.) | 0 | -0.56 (-1.38 to 0.26) | -0.50  (-1.07 to 0.06) |  |  | -2.19 (-2.79 to -1.59) |  |
| 2 | ψ (access)p(cont) | 0.27 | -0.34 (-1.30 to 0.61) | -0.58  (-1.21 to 0.04) |  |  | -2.37 (-3.01 to -1.74) | 0.26  (-0.05 to 0.57) |
| 3 | ψ (.)p(.) | 0.45 | -0.49 (-1.31 to 0.33) |  |  |  | -2.20 (-2.81 to -1.59) |  |
| 4 | ψ (.)p(cont) | 1.16 | -0.32 (-1.25 to 0.60) |  |  |  | -2.35 (-3.00 to -1.70) | 0.22  (-0.09 to 0.53) |
| 5 | ψ (access+forest)p(.) | 1.8 | -0.56 (-1.39 to 0.26) | -0.62  (-1.31 to 0.08) | 0.19  (-0.41 to 0.79) |  | -2.19 (-2.79 to -1.59) |  |
| 6 | ψ (access+prey)p(.) | 1.81 | -0.48 (-1.40 to 0.44) | -0.56  (-1.17 to 0.05) |  | 0.51  (-1.16 to 2.17) | -2.23 (-2.85 to -1.61) |  |
| **cumulative AIC weight** | | |  | 0.56 | 0.09 | 0.29 |  | 0.45 |
