# Supplementary material for: Effects of human impacts on habitat use, activity patterns and ecological relationships among medium and small felids of the Atlantic Forest
Source: PLoS One. 2018 Aug 1;13(8):e0200806. doi: 10.1371/journal.pone.0200806 (PMC6070200; doi:10.1371/journal.pone.0200806)
Supplement: S8 Table — Models were ordered according to the lowest value of AICc. Models were run at PRESENCE 6.2. (DOCX) [file pone.0200806.s009.docx]

S8 Table. **Co-ocurrence models for ocelot (species A) and for southern tiger cat (species B).** Models were ordered according to the lowest value of AICc. Models were run at PRESENCE 6.2.

| Model | no.Par. | AICc | ΔAICc | AICc wgt |
| --- | --- | --- | --- | --- |
| ψA, ψBA, ψBa, AccA, Land, pA,rA, pB=rBA=rBa | 9 | 564.81 | 0 | 0.46 |
| ψA, ψBA, ψBa, AccAB, Land, pA, rA, pB=rBA=rBa | 10 | 567.05 | 2.24 | 0.15 |
| ψA, ψBA, ψBa, AccA, LandA, pA, rA, pB=rBA=rBa | 9 | 568.23 | 3.42 | 0.08 |
| ψA, ψBA, ψBa, AccA, LandAB, pA, rA, pB=rBA=rBa | 11 | 568.54 | 3.73 | 0.07 |
| ψA, ψBA=ψBa, AccA, Land, pA,pB,rA,rBA=rBa | 9 | 568.88 | 4.07 | 0.06 |
| ψA, ψBA, ψBa, AccA, Land, pA,pB,rA,rBA,rBa | 11 | 569.29 | 4.48 | 0.05 |
| ψA, ψBA=ψBa, AccA, LandAB, pA, rA, pB=rBA=rBa | 10 | 569.87 | 5.06 | 0.04 |
| ψA, ψBA=ψBa, AccAB, Land, pA, rA, pB=rBA=rBa | 9 | 570.69 | 5.88 | 0.02 |
| ψA, ψBA=ψBa, AccA, Land, pA,pB,rA,rBA,rBa | 10 | 570.91 | 6.1 | 0.02 |
| ψA, ψBA, ψBa, AccAB, LandA, pA, rA, pB=rBA=rBa | 10 | 572.59 | 7.78 | 0.01 |
| ψA, ψBA, ψBa, AccA, pA, rA, pB=rBA=rBa | 7 | 573.05 | 8.24 | 0.01 |
| ψA, ψBA, ψBa, Acc, Land, pA, rA, pB, rBA=rBa | 10 | 573.21 | 8.4 | 0.01 |
| ψA, ψBA, ψBa, AccA, Land, pA, rA, pB,rBA=rBa | 10 | 573.21 | 8.4 | 0.01 |
| ψA, ψBA=ψBa, AccA, Land, pA, rA, pB=rBA=rBa | 8 | 573.44 | 8.63 | 0.01 |
| ψA, ψBA, ψBa, AccA, Land, pA=rA,pB=rBA=rBa | 8 | 575.47 | 10.66 | 0.00 |
| ψA, ψBA, ψBa, Acc, pA, rA, pB,rBA=rBa | 8 | 580.67 | 15.86 | 0.00 |
| ψA, ψBA, ψBa, Acc, pA, rA, pB=rBA=rBa | 7 | 582.4 | 17.59 | 0.00 |
| ψA, ψBA, ψBa, Acc, pA, rA, pB, rBA,rBa | 9 | 582.8 | 17.99 | 0.00 |
| ψA, ψBA=ψBa, pA, rA, pB, rBA=rBa | 6 | 598.53 | 33.72 | 0.00 |
| ψA, ψBA=ψBa, pA, rA, pB, rBA,rBa | 7 | 599.62 | 34.81 | 0.00 |
| ψA, ψBA, ψBa, pA, rA, pB, rBA=rBa | 7 | 600.7 | 35.89 | 0.00 |
| ψA, ψBA, ψBa, pA, rA, pB, rBA,rBa | 8 | 601.76 | 36.95 | 0.00 |

ψA= probability of occurrence for ocelots. ψBA= probability of occurrence for southern tiger cats given ocelots are present. ψBa= probability of occurrence for southern tiger cat given ocelots are absent. AccA= human access cost covariate for ocelots, AccAB= human access cost covariate for ocelots and southern tiger cats with different effect for each species, Acc= human access cost covariate for ocelots and southern tiger cats with the same effect for both species. Land= landscape condition for ocelots and southern tiger cats with the same effect for both species, LandAB= landscape condition for ocelots and southern tiger cats with different effect for each species, LandA= landscape condition for ocelots, pA= probability of detection for ocelots given southern tiger cats are absent, pB= probability of detection for southern tiger cats given ocelots are absent , rA= probability of detection for ocelots given both species are present, rBA= probability of detection for southern tiger cats given both species are present and ocelots are detected , rBa= probability of detection for southern tiger cats given both species are present and ocelots are not detected.
