# Supplementary material for: Effects of human impacts on habitat use, activity patterns and ecological relationships among medium and small felids of the Atlantic Forest
Source: PLoS One. 2018 Aug 1;13(8):e0200806. doi: 10.1371/journal.pone.0200806 (PMC6070200; doi:10.1371/journal.pone.0200806)
Supplement: S9 Table — (DOCX) [file pone.0200806.s010.docx]

S9 Table. **Beta estimates and their confidence intervals (CI 95%) for each parameter included in the best model of the co-occurrence models for ocelots and southern tiger cats.**

| Model | ψA | ψBA | ψBa | ψAccA | ψLand.CF | ψLand.FF | pA | pB | rA |
| --- | --- | --- | --- | --- | --- | --- | --- | --- | --- |
| 1 | -13.45  (-22.75 to  -4.14) | -7.66  (-12.60 to  -2.72) | 0.45  (-1.08 to 1.99) | 11.03  (3.45 to 18.61) | 7.52  (2.56 to 12.48) | 5.98  (1.11 to 10.84) | -3.50  (-4.21 to  -2.80) | -2.58  (-2.95 to  -2.21) | -1.48  (-1.96 to  -1.00) |

Model 1: ψA, ψBA, ψBa, AccA, Land, pA,rA, pB=rBA=rBa.
