# Supplementary material for: Effects of human impacts on habitat use, activity patterns and ecological relationships among medium and small felids of the Atlantic Forest
Source: PLoS One. 2018 Aug 1;13(8):e0200806. doi: 10.1371/journal.pone.0200806 (PMC6070200; doi:10.1371/journal.pone.0200806)
Supplement: S10 Table — (DOCX) [file pone.0200806.s011.docx]

S10 Table. **Mean probability of occurrence for southern tiger cats for the entire study area and discriminated by landscape condition (CF= continuous forest, FF= fragmented forest, PP= pine plantations).**

|  | **ψB** |
| --- | --- |
| **CF** | 0.50 (0.25- 0.76) |
| **FF** | 0.65 (0.49- 0.81) |
| **PP** | 0.43 (0.10- 0.76) |
| **Study area** | **0.53 (0.28- 0.78)** |
