# Supplementary material for: Effects of human impacts on habitat use, activity patterns and ecological relationships among medium and small felids of the Atlantic Forest
Source: PLoS One. 2018 Aug 1;13(8):e0200806. doi: 10.1371/journal.pone.0200806 (PMC6070200; doi:10.1371/journal.pone.0200806)
Supplement: S11 Table — (DOCX) [file pone.0200806.s012.docx]

S11 Table. Frequency of stations with low (0- 0.33), intermediate (0.34-0.66) and high (0.66-1) probability of southern tiger cat occurrence according to the landscape condition.

| **ψB** | **CF** | **FF** | **PP** |
| --- | --- | --- | --- |
| 0 – 0.33 | 1 | 21 | 19 |
| 0.34 – 0.66 | 48 | 10 | 43 |
| 0.66 - 1 | 4 | 38 | 0 |
